# Selective tubular activation of hypoxia-inducible factor-2α has dual effects on renal fibrosis

**DOI:** 10.1038/s41598-017-11829-2

**Published:** 2017-09-12

**Authors:** Kyoung Hye Kong, Hyung Jung Oh, Beom Jin Lim, Minsuk Kim, Ki-Hwan Han, Youn-Hee Choi, Kihwan Kwon, Bo Young Nam, Kyoung Sook Park, Jung Tak Park, Seung Hyeok Han, Tae-Hyun Yoo, Shina Lee, Seung-Jung Kim, Duk-Hee Kang, Kyu Bok Choi, Vera Eremina, Susan E. Quaggin, Dong-Ryeol Ryu, Shin-Wook Kang

**Affiliations:** 1 Graduate School, Ewha Womans University, Seoul, Korea; 2 Ewha Institute of Convergence Medicine, Ewha Womans University, Seoul, Korea; 3 College of Medicine, Yonsei University, Seoul, Korea; 4 School of Medicine, Ewha Womans University, Seoul, Korea; 5 Tissue Injury Defense Research Center, Ewha Womans University, Seoul, Korea; 6 The Samuel Lunenfeld Research Institute, Toronto, Ontario, Canada; 7 Feinberg Cardiovascular Research Institute and Division of Nephrology and Hypertension, Northwestern University, Chicago, Illinois USA

## Abstract

Hypoxia-inducible factor (HIF) is a key transcriptional factor in the response to hypoxia. Although the effect of HIF activation in chronic kidney disease (CKD) has been widely evaluated, the results have been inconsistent until now. This study aimed to investigate the effects of HIF-2α activation on renal fibrosis according to the activation timing in inducible tubule-specific transgenic mice with non-diabetic CKD. HIF-2α activation in renal tubular cells upregulated mRNA and protein expressions of fibronectin and type 1 collagen associated with the activation of p38 mitogen-activated protein kinase. In CKD mice, activation of HIF-2α at the beginning of CKD significantly aggravated renal fibrosis, whereas it did not lead to renal dysfunction. However, activation at a late-stage of CKD abrogated both renal dysfunction and fibrosis, which was associated with restoration of renal vasculature and amelioration of hypoxia through increased renal tubular expression of VEGF and its isoforms. As with tubular cells with HIF-2α activation, those under hypoxia also upregulated VEGF, fibronectin, and type 1 collagen expressions associated with HIF-1α activation. In conclusion, late-stage renal tubular HIF-2α activation has protective effects on renal fibrosis and the resultant renal dysfunction, thus it could represent a therapeutic target in late stage of CKD.

## Introduction

Regardless of the type of initial injury to the kidney, renal hypoxia is the common final pathway of renal fibrosis^1^, which is regarded a prime target for preventing the progression of chronic kidney disease (CKD) to end-stage renal disease.

Hypoxia-inducible factor (HIF) is a key transcriptional factor in the regulation of the adaptive response to hypoxia. HIF is a heterodimeric complex that has three forms (HIF-1, HIF-2, and HIF-3), which differ in their α-subunit. If the α-subunit is not hydroxylated by prolyl hydroxylase under hypoxic conditions, it cannot be recognized by von Hippel-Lindau tumor suppressor protein (pVHL), which is part of an E3-ubiquitin ligase complex that targets HIF-α for proteosomal degradation^2^. Then, α-subunit combines with a constitutively expressed β-subunit in the nucleus. Finally, HIF regulates the expression of salient target genes that are involved in numerous biological processes, including energy metabolism, angiogenesis, erythropoiesis, iron metabolism, cell proliferation, and apoptosis^2^.

HIF activation ameliorates tissue hypoxia and eventually helps the hypoxic cells survive. Even though the effect of HIF activation in CKD has been widely evaluated, the results have been inconsistent. Reduced renal fibrosis by genetic ablation of tubular HIF-1 suggested a profibrotic role of HIF^3^, while contrary results demonstrated that an HIF stabilizer exerted a beneficial effect on renal fibrosis in an animal model of CKD^4^.

Accumulating evidence suggests that HIFs exert beneficial effects in diabetic nephropathy. Activation of HIFs by cobalt chloride, a non-specific pan-HIF activator, attenuated diabetes-induced alteration in oxygen metabolism and mitochondrial leak respiration, proteinuria, and tubulointerstitial damage^5, 6^. However, in non-diabetic CKD, findings are largely conflicting. Renal fibrosis was aggravated in nephrectomized VHL^–/–^ mice^7^, in which HIFs are activated in the whole body from the embryonic stage, and it was abrogated in genetically HIF-1-ablated mice with unilateral ureteral obstruction (UUO)^3^, suggesting a profibrotic role of HIF. In contrast, cobalt chloride administration ameliorated cyclosporine A-induced afferent arteriolopathy and tubulointerstitial injury in the kidney^8^. Moreover, peritubular capillary networks were preserved in cobalt chloride-treated rats with remnant kidneys^9^.

In this study, we aimed to investigate the impact of tubular HIF-2α activation on renal fibrosis according to the timing of activation.

## Results

### Generation of mice with inducible renal tubular HIF-2α activation

We used a transgenic mouse line containing hemagglutinin (HA)-tagged HIF2dPA, which escapes from recognition by pVHL owing to a proline to alanine substitution, preceded by a floxed stop codon cassette, rendering HIF-2α expression Cre-dependent.

**Figure 1:**
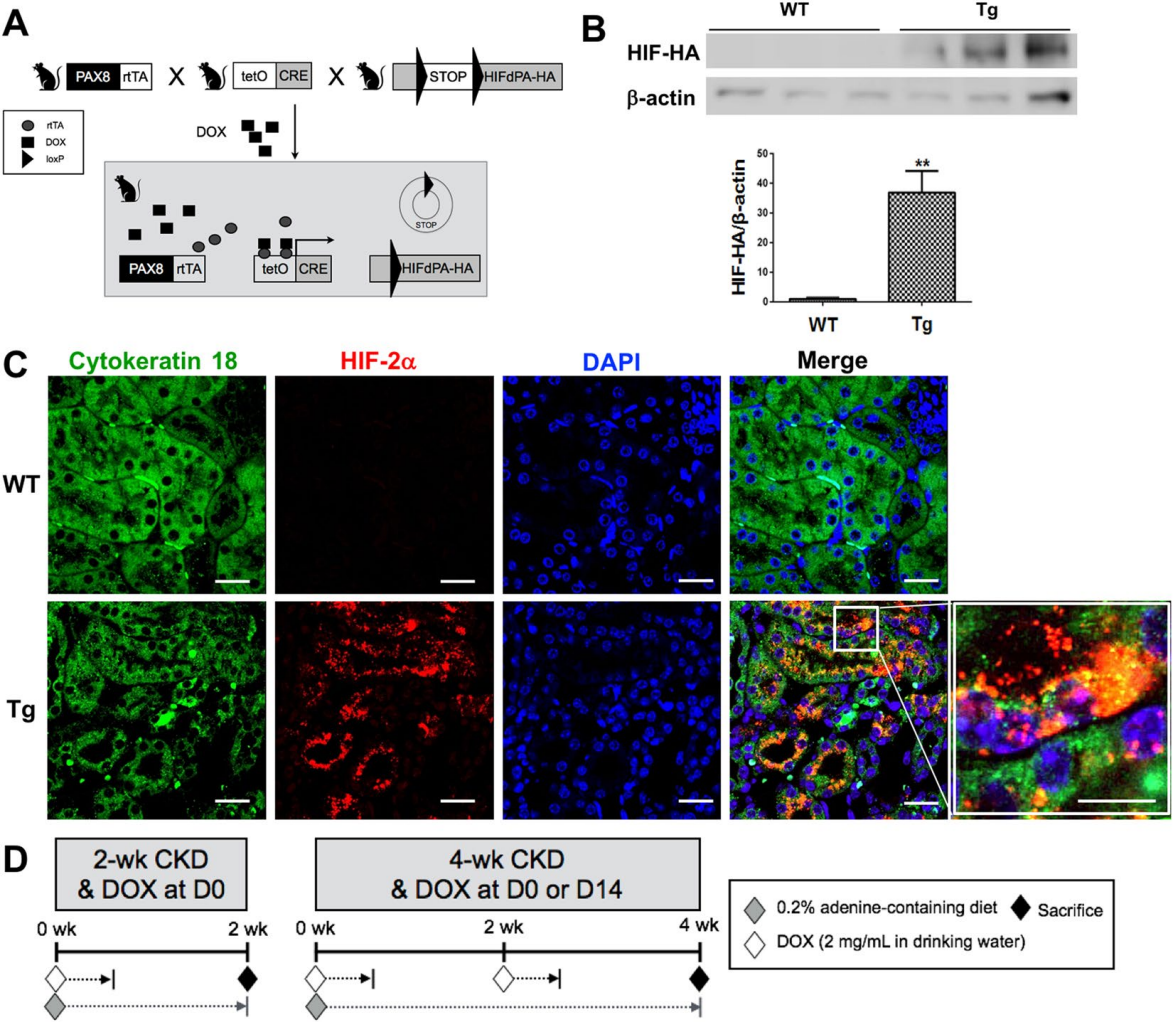
Breeding strategy and the experimental design. (**A**) Breeding strategy to make transgenic (Tg) mice in renal tubular cells. (**B**,**C**) Immunofluorescent staining and western blot analysis using anti-hemagglutin (HA) antibody (n = 3 per group) or anti-HIF-2α antibody (n = 4 per group). The cropped blots are displayed; full-length blots are presented in Supplementary Fig. 5. (**D**) Design for the timing effect of renal tubular selective HIF-2α activation. **P < 0.01 *vs*. WT mice; bar graphs show mean ± SEM. Scale bars, 20 μm.

To activate HIF-2α gene expression in renal tubular cells at the intended time, DOX has been added for 3 days and we bred HIF2dPA-HA mice^10^ with mice carrying tetO-Cre and Pax8-rtTA transgenes (Fig. 1A).

The induction of HIF-2α-HA protein expression in renal tubular cells was verified using by western blotting and immunofluorescent staining after doxycycline (DOX) administration. HIF-2α-HA protein was expressed in the transgenic mice, whereas it was hardly detected in the wild-type mice (Fig. 1B,C). The immunofluorescent signal of HIF-2α was observed in the nucleus as well as the cytoplasm of renal tubular cells (Fig. 1C). Figure 1D is the diagram depicting the scheme of animal experiments to investigate the impact of renal tubular HIF-2α activation on renal fibrosis according to the timing of HIF activation. Renal fibrosis and CKD was induced in wild-type and transgenic mice by feeding the mice a diet containing 0.2% adenine for 2 or 4 weeks. Mice fed with a conventional diet were used as controls.

### Renal dysfunction is ameliorated by late, but not by early HIF-2α activation in CKD mice model

There were no differences in body weight between wild-type and transgenic mice at both 2 weeks and 4 weeks (Table 1). Body weights of wild-type CKD mice tended to be lower without statistical significance compared to controls, whereas those of all transgenic CKD mice were significantly lower than those of controls. Body weights of transgenic mice at baseline before HIF-2α gene induction were lower than that of wild-type mice, and those were significantly decreased after CKD induction in both wild-type and transgenic mice, whereas those of transgenic mice with normal diet did not decrease (Supplementary Table 1).

**Table 1:** Animal data of control mice, and wild-type and HIF-2α transgenic CKD mice at 2 weeks and 4 weeks.

Parameters	Control	At 2 weeks		At 4 weeks		
		WT	Tg	WT	Tg (DOX at D0)	Tg (DOX at D14)
Body Weight (g)	26.7 ± 1.1	23.5 ± 0.9	22.3 ± 0.8^*^	23.5 ± 1.3	21.3 ± 0.8^*^	20.9 ± 1.0^**^
BUN (mg/dL)	28.3 ± 1.6	85.6 ± 11.4^***^	74.3 ± 5.6^***^	102.9 ± 8.7^***^	91.9 ± 7.5^***^	73.0 ± 7.5^***, ##^
Serum creatinine (mg/dL)	0.25 ± 0.01	0.61 ± 0.07^***^	0.56 ± 0.05^***^	0.79 ± 0.05^***, ††^	0.71 ± 0.05^***^	0.63 ± 0.02^***, #^

CKD, chronic kidney disease; BUN, blood urea nitrogen; WT, wild-type; Tg, transgenic; DOX, doxycycline.

*P < 0.05, **P < 0.01, and ***P < 0.001, and *vs*. controls; ^#^ *P* < 0.05, and ^##^ *P* < 0.01 *vs*. WT mice at 4 weeks; ^††^ *P* < 0.01 *vs*. WT mice at 2 weeks.

Blood urea nitrogen (BUN) levels were significantly higher in CKD mice at 2 weeks (85.6 ± 11.4 mg/dL) and at 4 weeks (102.9 ± 8.7 mg/dL) than in control mice (28.3 ± 1.6 mg/dL) (*P* < 0.001 and *P* < 0.0001, respectively). Similarly, serum creatinine (Cr) concentrations were significantly higher in CKD mice at 2 weeks (0.61 ± 0.07 mg/dL) and 4 weeks (0.79 ± 0.05 mg/dL) than in the controls (0.25 ± 0.01 mg/dL) (*P* < 0.001 and *P* < 0.001, respectively). At 2 weeks, transgenic CKD mice with HIF-2α activation from the beginning of the 0.2% adenine diet showed BUN (74.3 ± 5.6 mg/dL) and Cr levels (0.56 ± 0.05 mg/dL) comparable to those of wild-type CKD mice (Table 1). Similarly, at 4 weeks after simultaneous HIF-2α activation and CKD induction, there were no significant differences in BUN (91.9 ± 7.5 mg/dL) and Cr concentrations (0.71 ± 0.05 mg/dL) between wild-type and transgenic CKD mice. In contrast, HIF-2α activation after 2 weeks of CKD induction significantly attenuated the increases in the BUN (73.0 ± 7.5 mg/dL, *P* < 0.01) and Cr levels (0.63 ± 0.02 mg/dL, *P* < 0.05) compared to wild-type CKD mice at 4 weeks.

### Tubular HIF-2α activation aggravates renal fibrosis at the initial stage of CKD, but inhibits fibrosis progression at an advanced stage of CKD

**Figure 2:**
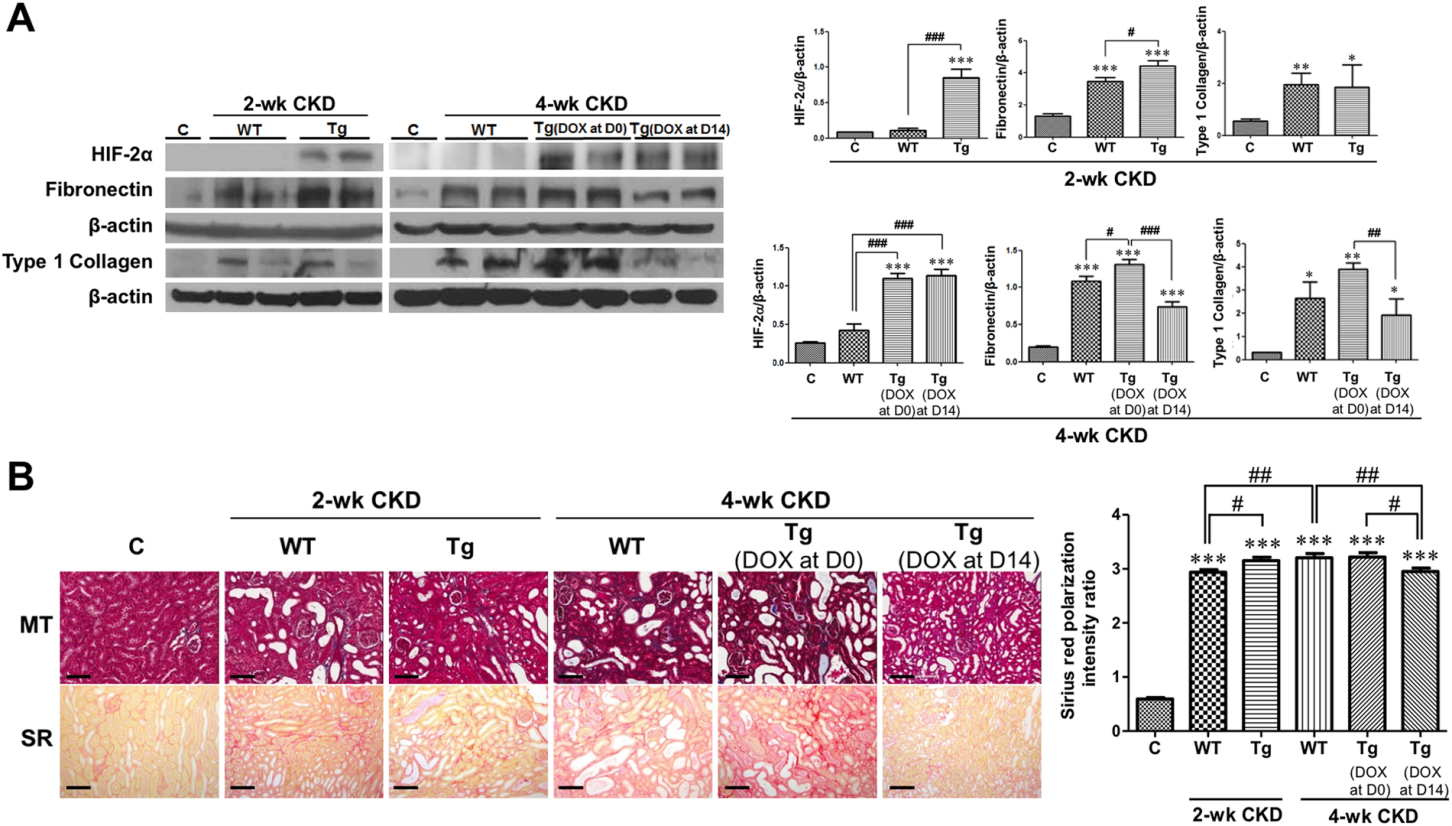
Selective renal tubular HIF-2α activation has dual effects on renal fibrosis. (**A**) Western blot results (n = 3 for control, n = 6 for other groups). Representative images of 3 repeated western blot analysis and quantification. (**B**) Representative images of similar sections showing Masson’s trichrome and Sirius red stainings. Sirius red-stained sections were viewed with polarization contrast illumination, converted into gray-scale images, and quantified using non-overlapping 10 × images over the entire kidney section (10 images per mouse, n = 2 for control, n = 7 for WT 2-week CKD group, n = 6 for other groups). The cropped blots are displayed; full-length blots are presented in Supplementary Fig. 5. *P < 0.05, **P < 0.01, and ***P < 0.001 *vs*. controls; ^#^ *P* < 0.05, ^##^ *P* < 0.01, and ^###^ *P* < 0.001 between the two groups; bar graphs show mean ± SEM. Scale bars, 100 μm.

We confirmed whether DOX-induced HIF-2α activation modified tubular HIF-2α protein expression or not. HIF-2α expression in the tubular cells of transgenic mice was significantly higher than that of both controls and corresponding wild-type mice by DOX administration (Fig. 2A and Supplementary Fig. 1).

Next, we investigated whether the timing of HIF-2α activation had differential effects on renal fibrosis by examining relevant protein expression and renal histology. Fibronectin protein expression was significantly higher in wild-type CKD mice at both 2 weeks and 4 weeks than in control mice (*P* < 0.001 and *P* < 0.001, respectively) (Fig. 2A). In addition, compared to wild-type CKD mice, the protein expression of fibronectin was 1.28-fold higher at 2 weeks (*P* < 0.05) and 1.20-fold higher at 4 weeks (*P* < 0.05) in transgenic CKD mice after simultaneous CKD induction and HIF-2α activation. However, when HIF-2α was activated at 2 weeks after CKD induction, the increase in fibronectin protein expression at 4 weeks was significantly abrogated by 31.6% (*P* < 0.001) as compared to wild-type CKD mice. Type 1 collagen protein expression was changed in a similar way.

The western blot results for fibronectin were confirmed by Masson’s trichrome and Sirius red staining (Fig. 2B). Quantification of renal fibrosis using gray-scale images of Sirius red staining captured under a polarized microscope revealed that renal fibrosis in wild-type CKD mice at 4 weeks was significantly ameliorated by HIF-2α activation at a later period but not by HIF-2α activation from the beginning of CKD induction (Fig. 2B and Supplementary Fig. 2).

### Later renal tubular HIF-2α activation restores reduced renal vasculature in CKD mice and consequently improves the level of hypoxia

**Figure 3:**
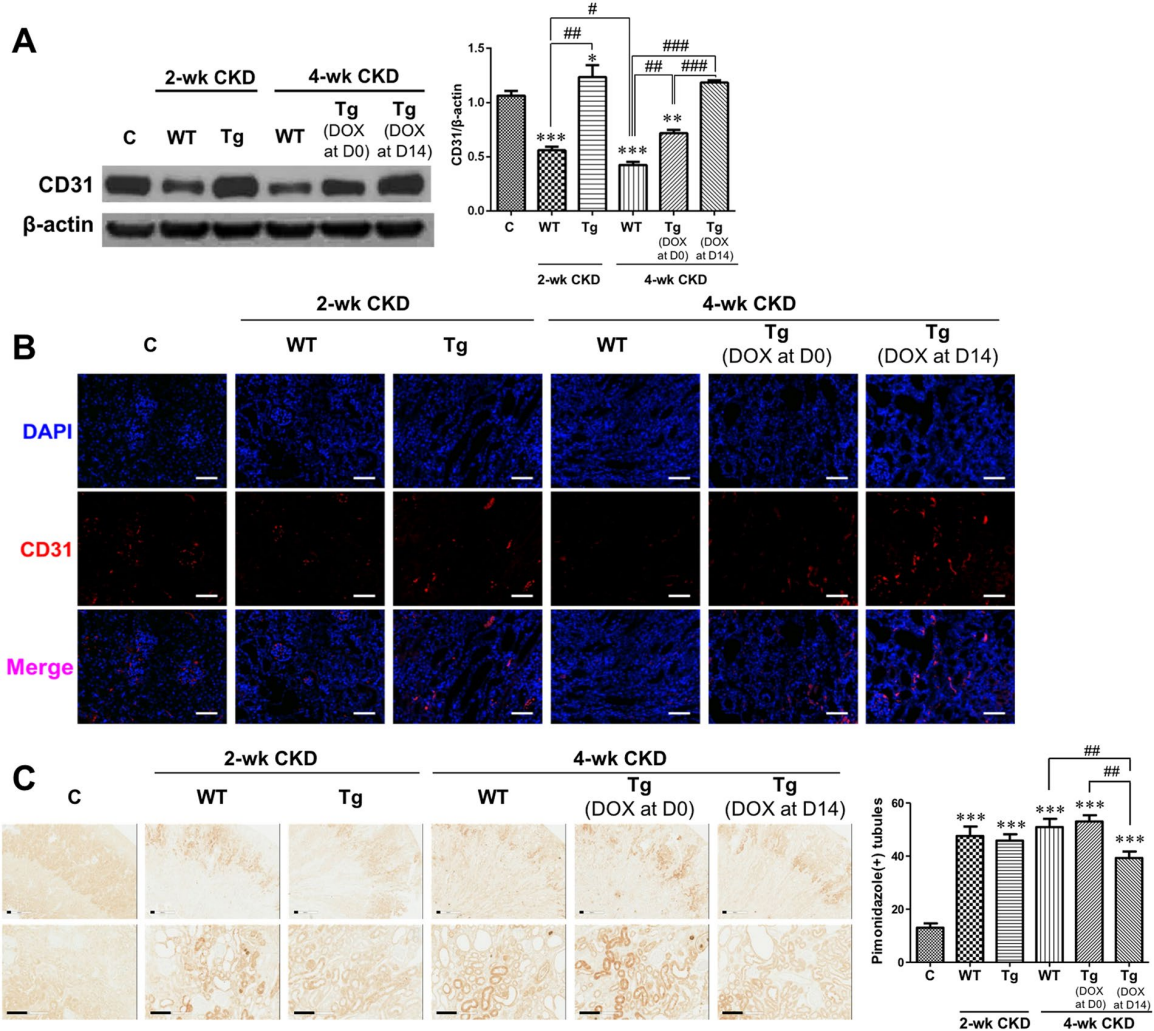
Renal vasculature and hypoxia were ameliorated with renal tubular HIF-2α activation. (**A**,**B**) Representative blot of 3 repeated western blot analysis for CD31 and representative images of immunofluorescent staining for CD31 (n = 3). (**C**) Representative images of immunohistochemical staining using pimonidazole (5 images per mouse, n = 2). The cropped blots are displayed; full-length blots are presented in Supplementary Fig. 5. *P < 0.05, **P < 0.01 and ***P < 0.001 *vs*. controls; ^#^ *P* < 0.05, ^##^ *P* < 0.01, and ^###^ *P* < 0.001 between the two groups; bar graphs show mean ± SEM. Scale bars, (**B**) 1000 μm and (**C**) 100 μm.

Western blot and immunofluorescent staining for the endothelial marker protein CD31 revealed that its expression in the kidneys at 2 weeks was significantly lower in wild-type CKD mice than in control mice, whereas it was significantly higher in HIF-2α transgenic CKD mice than in wild-type CKD mice (Fig. 3A,B). However, at 4 weeks of CKD, renal CD31 protein expression depended on the timing of HIF-2α transgene induction; it was significantly higher in transgenic CKD mice with later HIF-2α activation than in control mice as well as transgenic CKD mice with HIF-2α activation from the beginning of CKD induction.

To identify renal hypoxia in CKD mice, we performed immunohistochemical staining for pimonidazole, a hypoxia marker. In the kidneys of CKD mice, immunoreactivity for pimonidazole was significantly greater than in control mice at both 2 and 4 weeks (Fig. 3C). There was no significant difference in immunoreactivity between wild-type and 2-week or 4-week transgenic CKD mice with HIF-2α activation from the beginning of CKD induction. However, the increase in pimonidazole-positive tubules at 4 weeks of CKD was significantly abrogated when HIF-2α was activated at a later period.

The mRNA expression of total vascular endothelial growth factor (VEGF) was significantly lower in wild-type CKD mice than that in controls at both 2 and 4 weeks (Supplementary Fig. 3). However, it was significantly higher in HIF-2α transgenic CKD mice than that in wild-type CKD mice at both 2 and 4 weeks, and there was more significant increase in transgenic mice with later HIF-2α activation than those of all other groups. The mRNA expressions of VEGF 120, 164, and 188 isoforms were lower in wild-type and HIF-2α transgenic CKD mice compared to controls at 2 weeks, whereas the decrement in mRNA expressions of VEGF isoforms in wild-type CKD mice significantly increased in HIF-2α transgenic CKD mice without regard to the activation timing at 4 weeks.

### HIF-2α overexpression affects the expression of various genes in isolated primary TECs

**Figure 4:**
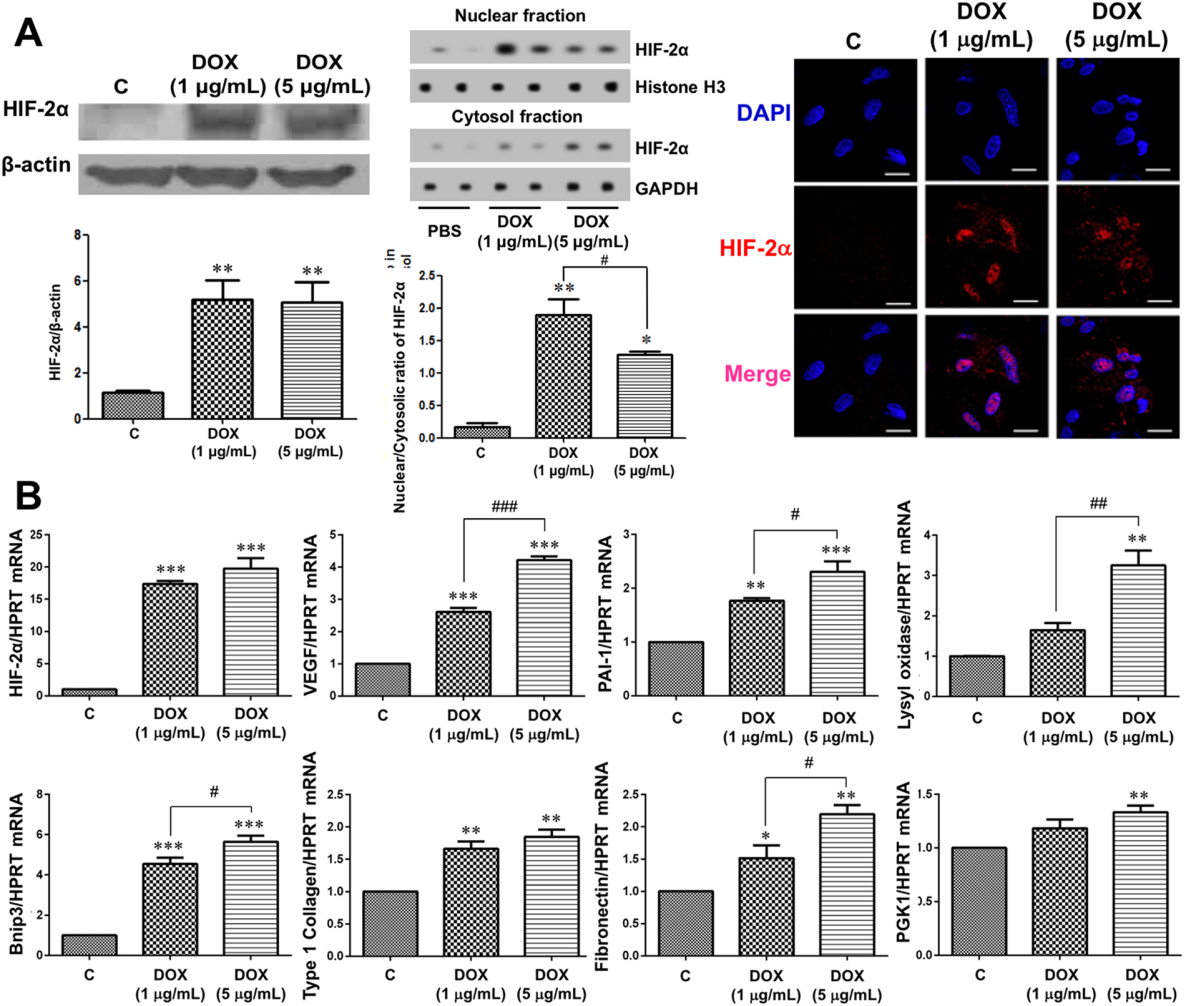
HIF-2α activation affects expression of various genes in cultured primary tubular epithelial cells (TECs). (**A**) After isolation of TECs from transgenic mice, doxycycline (DOX) was added to the media or not (control), and HIF-2α induction in TECs by DOX was confirmed by western blotting and immunofluorescent staining (n = 3). (**B**) In RT-qPCR for the mRNA (n = 3). qPCRs were performed 3 to 4 times with RNA isolated lysates derived from 3 independent cell culture experiments. The cropped blots are displayed; full-length blots are presented in Supplementary Fig. 5. *P < 0.05, **P < 0.01, and ***P < 0.001 *vs*. controls; ^#^ *P* < 0.05, ^##^ *P* < 0.01, and ^###^ *P* < 0.001 between the two groups; bar graphs show mean ± SEM. Scale bars, 20 μm.

After isolation of TECs from transgenic mice, DOX was added in the media at a final concentration of 1 μg/mL or 5 μg/mL for transgene induction. We demonstrated that there was no significant cytotoxicity at these concentrations of DOX by the MTS assay (data not shown). TECs cultured in the media with DOX showed significantly higher HIF-2α protein expression in western blotting and immunofluorescent staining, and HIF-2α migrated into the nucleus at both concentrations of DOX. Moreover, the ratios in cultured cells with 1 μg/mL and 5 μg/mL DOX supplementation were significantly higher compared to control (Fig. 4A).

Quantitative reverse transcription polymerase chain reaction (RT-qPCR) analysis showed that the mRNA expression of HIF-2α, VEGF, plasminogen activator inhibitor-1 (PAI-1), BCL2/adenovirus E1B 19 kDa-interacting protein 3 (Bnip3), type 1 collagen, and fibronectin was significantly higher in TECs treated with DOX at both concentrations than in untreated control TECs (Fig. 4B). Lysyl oxidase and phosphoglycerate kinase 1 (PGK1) mRNA expression were significantly higher in TECs treated with DOX at 5 μg/mL but not at 1 μg/mL.

### HIF-2α overexpression in isolated primary TECs affects the expression of various proteins in a time-dependent way

**Figure 5:**
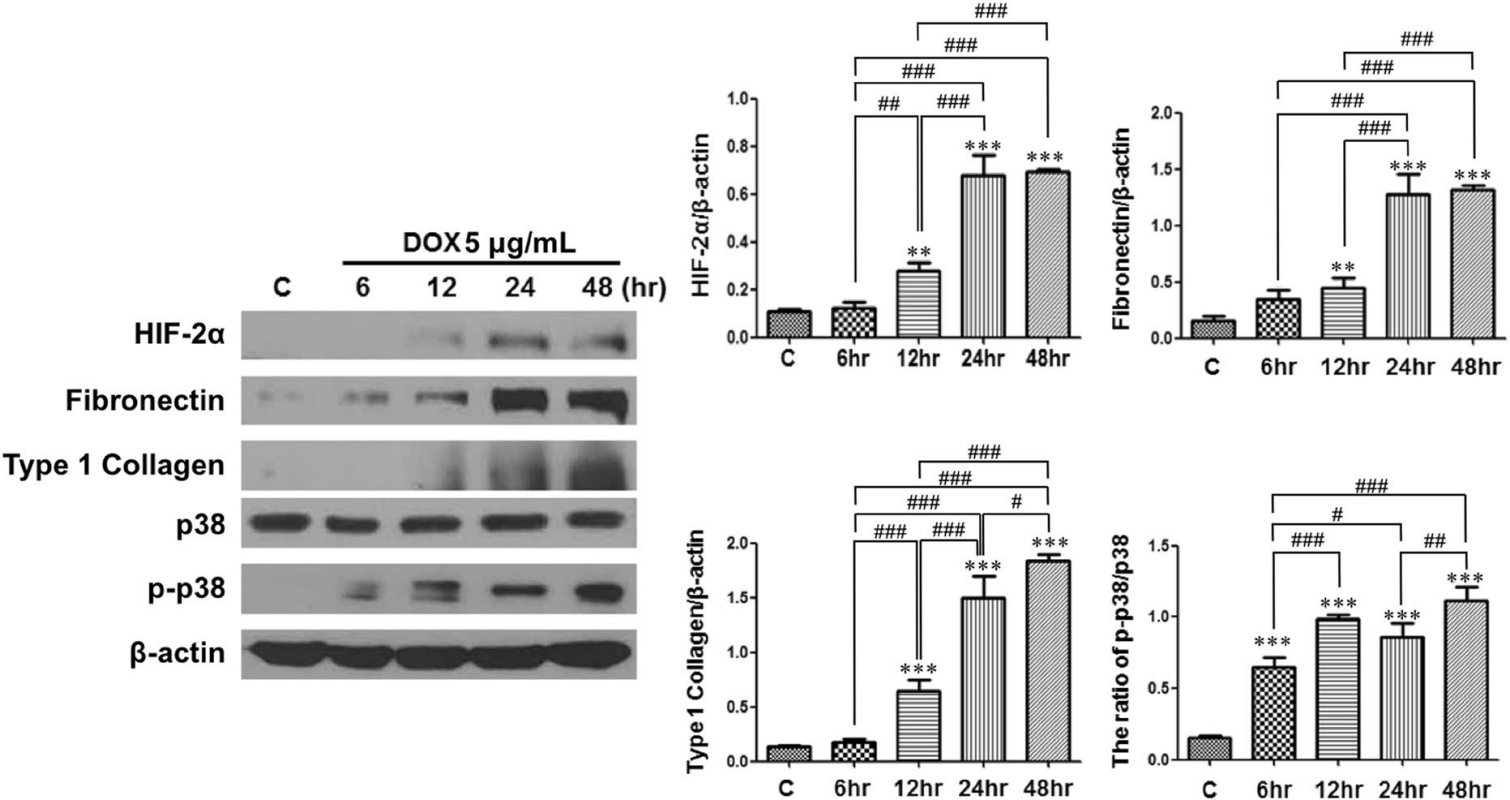
HIF-2α overexpression in isolated primary tubular epithelial cells (TECs) affects the expression of various proteins in a time-dependent way. Representative images of 3 repeated western blot analysis and quantification (n = 3). The cropped blots are displayed; full-length blots are presented in Supplementary Fig. 5. **P < 0.01 and ***P < 0.001 *vs*. controls; ^#^ *P* < 0.05, ^##^ *P* < 0.01, and ^###^ *P* < 0.001 between the two groups; bar graphs show mean ± SEM.

HIF-2α protein expression significantly increased as the DOX-treated time increased. According to the increase in HIF-2α protein expression, the protein expressions of fibronectin and type 1 collagen were also significantly upregulated in a time-dependent way. The ratio of p-p38/p38 was elevated in DOX-treated groups compared with control (Fig. 5).

### TECs under hypoxia also induced fibronectin and type 1 collagen like HIF-2α- overexpressed TECs, but not via HIF-2α expression

**Figure 6:**
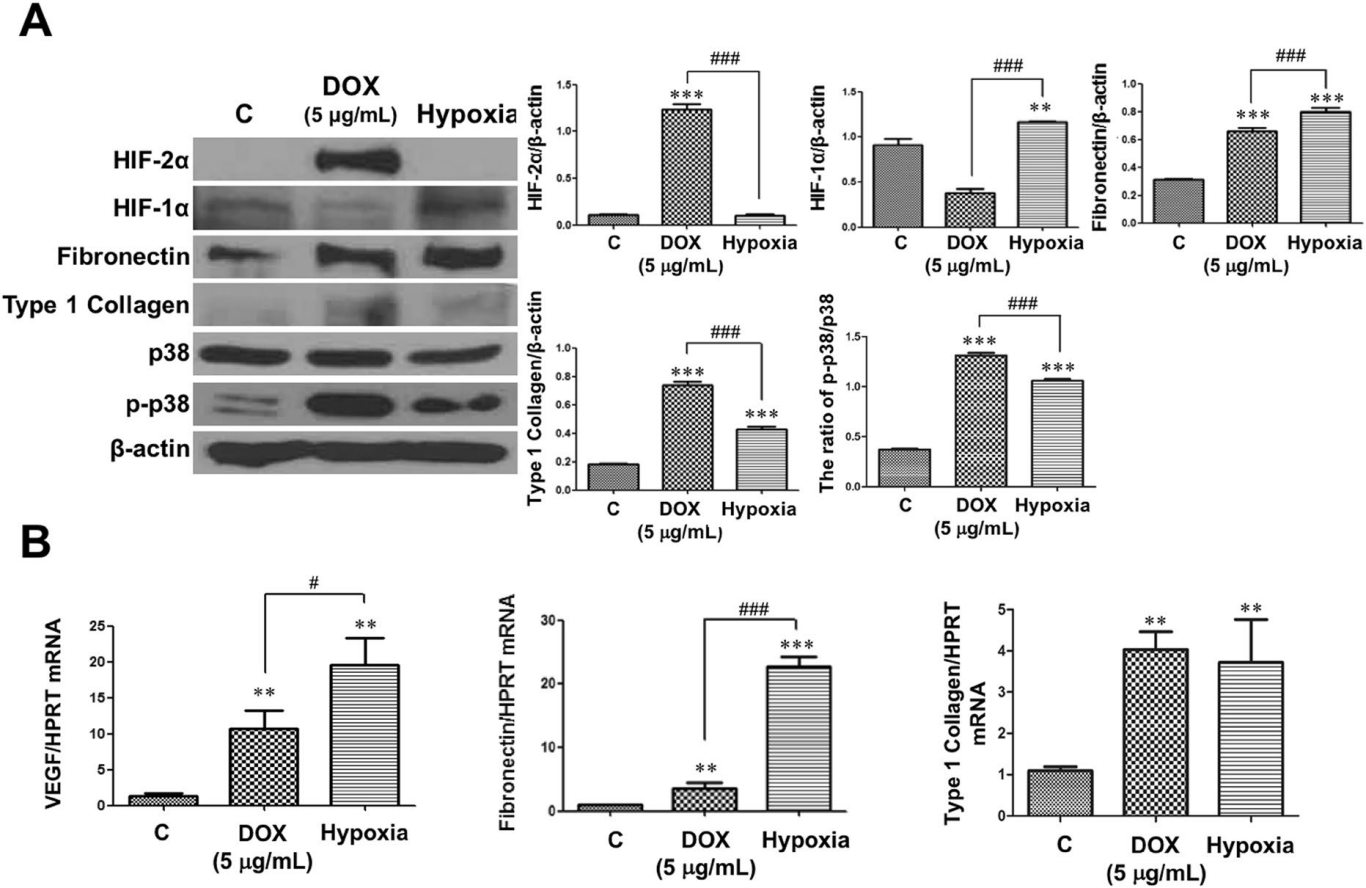
Protein and mRNA expressions changed similarly between doxycycline (DOX)-treated and hypoxia-exposed tubular epithelial cells (TECs). Representative images of 3 repeated western blot analysis and quantification (n = 3). qPCRs were performed 3 to 4 times with RNA isolated lysates derived from 3 independent cell culture experiments. The cropped blots are displayed; full-length blots are presented in Supplementary Fig. 5. **P < 0.01, and ***P < 0.001 *vs*. controls; ^#^ *P* < 0.05, ^##^ *P* < 0.01, and ^###^ *P* < 0.001 between the two groups; bar graphs show mean ± SEM.

HIF-2α protein expression did not increase in hypoxic TECs compared with control cells (Fig. 6A). However, HIF-1α expression significantly increased in hypoxic TECs compared to controls. Nevertheless, protein expressions of fibronectin and type 1 collagen were higher in both of DOX-treated and hypoxia-exposed TECs than those in controls. In addition, VEGF, fibronectin, and type 1 collagen mRNA expressions were higher in both groups than those in controls (Fig. 6B). VEGF and fibronectin mRNA expressions were much higher in hypoxia-exposed TECs than those in DOX-treated TECs.

### Human IgA nephropathy patients show distinct gene expression patterns according to the stage of tubular atrophy/interstitial fibrosis

Finally, we performed RT-qPCR analysis of a variety of relevant genes in microdissected renal tubulointerstitium of patients with IgA nephropathy. The subjects were divided according to the stage of tubular atrophy/interstitial fibrosis (T stage) by the Oxford classification. Renal HIF-2α, VEGF, fibronectin, and TGF-β mRNA expression was significantly higher in stage T1 than in stages T0 and T2. In contrast, HIF-1α mRNA expression was significantly lower in stage T1 patients than in stage T2 subjects. CTGF expression did not differ among the three groups (Supplementary Fig. 4A).

There were no significant differences in the gene expression patterns in the groups stratified by the CKD stage based on estimated glomerular filtration rate (eGFR) (Supplementary Fig. 4B).

## Discussion

In this study, we found that renal tubular HIF-2α activation had dual effects on renal fibrosis according to the degree of tubulointerstitial hypoxia in a non-diabetic CKD model. When HIF-2α expression was induced at initiation of CKD, it played a predominantly profibrotic role. In contrast, HIF-2α activation at a later stage of CKD protected the kidney from the progression of renal fibrosis, resulting in functional preservation.

Until now, some studies have explored the effects of individual HIFs on renal fibrosis, but it is not conclusive yet. Global activation of HIF suppressed inflammation and fibrogenesis in UUO mice^11^. However, tubular cell-specific HIF-1 deletion using HIF-1α^2lox/2lox^:PEPCK-cre mice inhibited the development of renal tubulointerstitial fibrosis in UUO mice^3^. In addition, continuous transgenic expression of HIF-2α by the Ksp-Cadherin promotor led to renal fibrosis and insufficiency, next to multiple renal cysts^12^. Administration of a prolyl hydroxylase inhibitor induced HIF-1α at an early stage in a rat remnant kidney model, while HIF-2α expression was increased at moderately advanced stages^4^.

Although HIF-1α and HIF-2α are closely related and show 48% overall amino acid identity, they have distinct but partially overlapping functions^10, 13^. Therefore, it is likely that the effects of HIF activation on renal fibrosis in CKD may be different according to the HIF class. In response to hypoxia, proximal TECs stabilized HIF-1α, but not HIF-2α^14^. Moreover, HIF-1α and HIF-2α expression may be differential in intermittent hypoxia (IH)-associated disease; HIF-1α protein expression is induced during intermittent hypoxia (IH)^15, 16^, while HIF-2α expression is repressed by IH through calpain-dependent mechanisms^17^. In addition, whereas HIF-1α was inducible in physiological renal mouse, rat and human tubular epithelia, HIF-2α was never detected in these cells in any species, and tubular cells required VHL inactivation to allow HIF-2α expression^12^. Recently, however, cobalt chloride treatment increased HIF-2α as well as HIF-1α protein expression in HK-2 cells, and renal HIF-1α and HIF-2α protein expression was simultaneously upregulated with L-mimosine administration after ischemia-reperfusion injury^18^. If HIF-1α and HIF-2α expressions are induced in human renal tubular cells by non-specific HIF stabilizers, the results of this study may have the clinical relevance. In addition, we found that TECs under hypoxia upregulated VEGF as well as fibronectin and type 1 collagen expressions as with tubular cells with HIF-2α activation. Since HIF-2α expression was hardly detected in hypoxia-exposed TECs, we assume that these effects were related to HIF-1α activation by hypoxic stimuli. However, both profibrotic and antihypoxic effect by HIF activation in TECs might be similar between HIF-1α and HIF-2α in case that HIF-1α or HIF-2α maintained in tubule cells at a sufficient concentration and time.

To our knowledge, this study is the first to investigate the consequences of tubular HIF-2α-selective activation using inducible tubule-specific transgenic mice. Tubular HIF-2α activation was directly associated with changes in renal function and tubulointerstitial fibrosis in CKD mice. In addition, the effect HIF-2α was different according to the activation timing in CKD. Administration of cobalt chloride for 1 week from the initiation of an ablation/infarction kidney model significantly reduced metabolic and hemodynamic abnormalities^19^. However, we surmised that HIF activation might be more beneficial at a later stage of CKD, because renal hypoxia can be worsened as CKD is progressive. In the present study, CKD mice had a significantly higher degree of renal hypoxia than control mice, and HIF-2α activation at a later stage of CKD mitigated renal hypoxia and abrogated renal dysfunction as well as the progression of fibrosis.

A previous report showed that HIF activation was not sufficient to overcome the hypoxic insult and did not sustain during the whole hypoxic period in longstanding CKD^20^. Therefore, renal cells cannot survive the hypoxia because of inadequate HIF stimuli in CKD. In fact, HIF-α activation using a prolyl hydroxylase inhibitor at a mid-advanced stage of CKD, but not at the early stage, resulted in an improvement in renal fibrosis and dysfunction^4^. In contrast, one study demonstrated that the effect of cobalt chloride on tubulointerstitial fibrosis in nephrectomized rats did not differ according to the timing of its administration^9^. The discrepancy between the findings in these two studies may in part be attributed to differences in the drugs used for HIF activation/stabilization, research design, or the timing of HIF induction. In the current study, the timing of HIF-2α activation was a prime determinant of the phenotypic changes in the kidneys; renal fibrosis was aggravated when HIF-2α was activated from the beginning of CKD, while it was ameliorated via an improvement in renal hypoxia when HIF-2α was activated at a later stage of CKD. Accordingly, stabilization of both HIF1 and HIF2 in a 5/6 renal ablation model of VHL^–/–^ mice was also associated with exacerbated renal fibrosis, and treatment with YC-1, an anti-HIF-1 agent, inhibited the progression of renal fibrosis in UUO model mice^7^. As VHL^–/–^ mice likely expressed a high level of HIF when CKD was generated, a high HIF level at the initiation of renal fibrosis might induce the profibrotic pathway cascade. In addition, whole-body deletion of VHL induces HIF activation in all types of renal cells, and thus, specific consequences of HIF activation in the kidney might be masked. In fact, HIF-2α activation in podocytes resulted in crescentic glomerulonephritis^21^.

A PAX8 promoter was used in this study to confine HIF-2α activation within renal tubular cells, which are a main source of VEGF in the tubulointerstitium^22^. Decreased tubular VEGF expression is significantly associated with the severity of peritubular capillary loss^19^. Furthermore, peritubular capillary density is positively correlated with proximal tubular size and negatively with interstitial volume in the human kidney, suggesting that ischemia induced by peritubular capillary loss could exacerbate tubulointerstitial injury^23^. In our study, cultured TECs with activated HIF-2α had higher VEGF expression than control cells. Moreover, we found *in vivo* that VEGF mRNA expressions consistently decreased in wild-type CKD mice compared to controls after CKD induction. In contrast with the inconsistent results in mRNA expressions of transgenic CKD mice at 2 weeks between total VEGF and its isoform 120, 164, and 188, which are known to play a major role on angiogenesis in the kidney^24, 25^, those were significantly higher in HIF-2α transgenic mice at 4 weeks of CKD induction. Of note, they were the highest in transgenic mice with later HIF-2α activation among all groups. We suppose that VEGF mRNA expression in tubular cells may be affected not only by HIF-2α activation but also by the degree of tubulointerstitial hypoxia.

In parallel with the pattern of VEGF expression, peritubular vasculature as assessed by CD31 expression was not diminished in transgenic CKD mice with later HIF-2α activation compared with control. Based on these findings, we postulate that HIF-2α activation at a later stage can lead to sustained release of VEGF, resulting in maintenance of vascular integrity and attenuation of tubulointerstitial hypoxia.

HIF-2α activation also was associated with an increase in the mRNA expression of PGK1, which plays a key role in coordinating glycolytic energy production with one-carbon metabolism, serine biosynthesis, and cellular redox regulation^26^, collectively promoting tubular cells to survive under hypoxic conditions. Meanwhile, HIF-2α activation in cultured TECs also induced profibrotic and proapoptotic genes, including fibronectin, type 1 collagen, PAI-1, lysyl oxidase, and Bnip3, indicating that they are under the control of HIF-2α. Similarly, HIF-2α activation from the initiation of CKD induced renal fibronectin and type 1 collagen expression at 2 and 4 weeks *in vivo*. In contrast, expressions of profibrotic genes along with renal dysfunction at 4 weeks were abrogated by HIF-2α activation at a later stage of CKD.

The exact underlying mechanism of selective tubular HIF-2α activation on renal fibrosis is inconclusive. Since the relationship between kidney fibrosis and mitogen-activated protein kinase (MAPK) pathway is well established^27^, we first evaluated p38 MAPK activation in TECs with HIF-2α activation, and found that phosphorylation of p38 MAPK was significantly associated with HIF-2α activation in TECs, suggesting that stimulation of p38 MAPK pathway by HIF-2α activation is, at least in part, could be related to the induction of renal fibrosis.

Tubular cell is not the main contributor to the progression of renal fibrosis. Renal fibrosis is induced by the tubular cells, fibroblasts, pericytes, endothelial cells, and even bone marrow-derived cells^28^. In this study, renal fibrosis induced by tubular HIF-2α activation did not lead renal dysfunction. Although HIF-2α activation in TECs significantly increased fibronectin and type 1 collagen expressions *in vitro* and early tubular HIF-2α overexpression in CKD mice increased their expressions *in vivo*, it was not associated with the deterioration in renal function assessed by BUN and creatinine level. Therefore, we suggest that profibrotic effects of HIF-2α activation in tubular cells is likely to be minor in the CKD progression.

**Figure 7:**
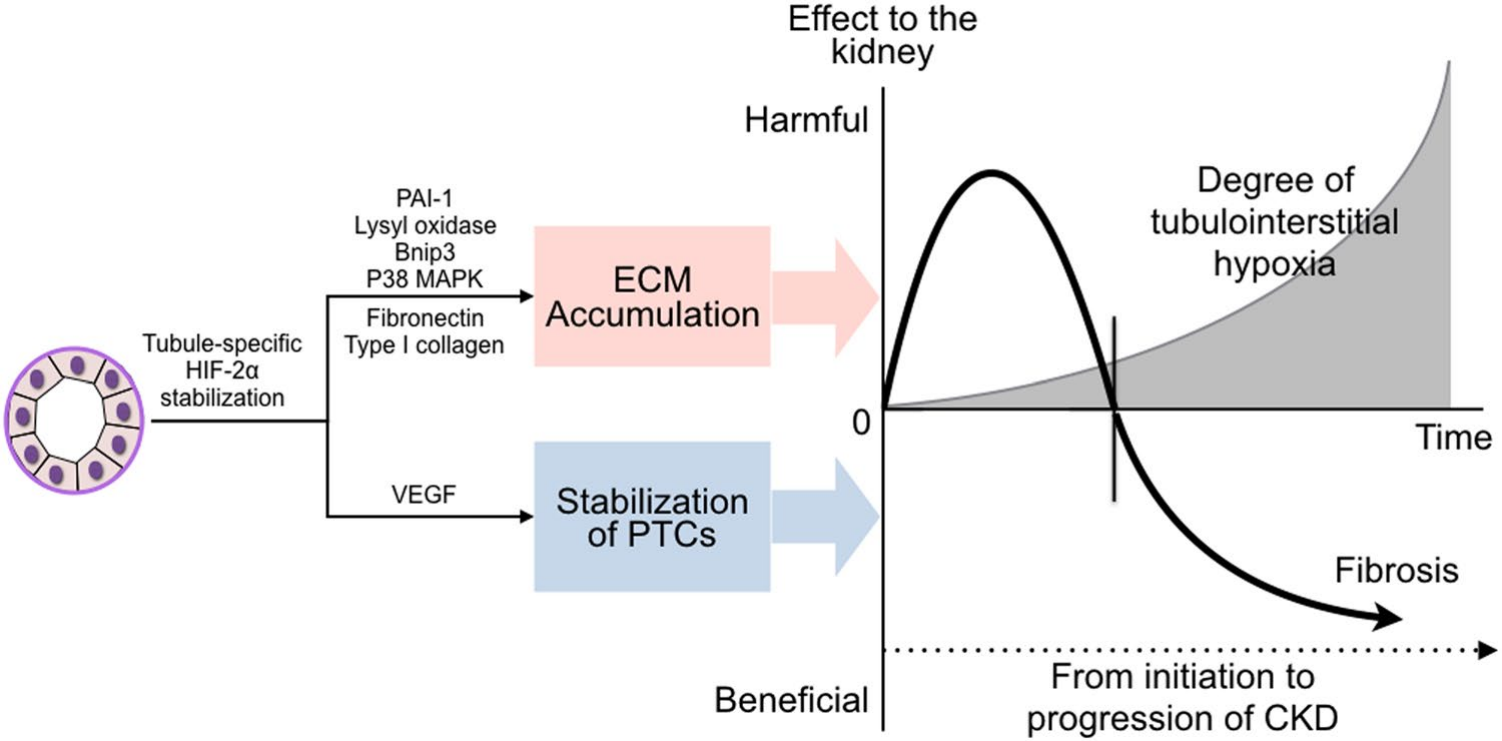
Schematic diagram for the dual effect of HIF-2α on renal fibrosis in chronic kidney disease (CKD). Net effect of tubule-specific stabilization of HIF-2α to the kidney is dependent on the degree of tubulointerstitial hypoxia. Although HIF-2α stimulation activates both antihypoxic pathway and profibrotic pathway, its net effect on renal fibrosis and the resultant renal dysfunction is different according to activated timing of HIF-2α. ECM, extracellular matrix; PTCs, peritubular capillaries.

Sustained renal tubular HIF-2α expression must have detrimental effects^29^, which accords closely with our findings. However, as aforementioned, HIF-2α activation at a later stage of CKD may reverse the deleterious impact of renal hypoxia by improvement of peritubular vascular integrity, and the positive effect may overcome the induction of profibrotic and proapoptotic genes by HIF-2α in tubule cells *per se* (Fig. 7). We need to consider overall effects of tubular HIF-2α activation on renal fibrosis and renal dysfunction in a body as a whole at a specific time.

Finally, we preliminarily investigated mRNA expression in the kidneys of IgA nephropathy patients according to the degree of tubular atrophy/interstitial fibrosis based on the Oxford classification. Interestingly, at stage T1, which is considered an early stage of renal fibrosis, the expression of multiple genes significantly differed from that at stages T0 and T2. Expression of HIF-2α, fibronectin, and TGF-β was significantly increased at stage T1. However, at stage T2, HIF-2α mRNA upregulation was not sustained, which might have led to a decrease in VEGF mRNA expression. Based on these findings, we inferred that a sustained release of VEGF at a later stage of renal fibrosis via HIF-2α stabilization could relieve tubulointerstitial hypoxia and inhibit the progression of fibrosis. However, the optimal timing for HIF-2α stabilization for protection against renal fibrosis should be further elucidated. HIF-2α and VEGF mRNA levels were significantly correlated with the stage of tubular atrophy/interstitial fibrosis but not with the eGFR stage. Therefore, it would remain difficult to decide on the proper timing for HIF-2α stabilization in clinical practice. In addition, although it has been recently reported that hypoxic HIF-2α protein expression can be transcriptionally regulated via insulin-like growth factor-II in some tumor cell lines^30, 31^, the expression of HIF-α subunits depends on the post-translational process. Therefore, further studies are necessary to elucidate whether the change in HIF-2α mRNA expression in the human kidney would result in altered protein expression of HIF-2α and phenotypic change.

There are some limitations in this study. First, we induced renal fibrosis and CKD by 0.2% adenine-containing diet. This CKD model was verified by other investigators. Administration of 0.2% adenine diet increased BUN and serum creatinine levels, and induced renal fibrosis and tissue hypoxia^32, 33^. However, it may not be the classic CKD model as 5/6 nephrectomized animal and we cannot totally exclude the chemical effect of adenine on our findings. Second, HIF-2α protein expression in kidneys was not significantly higher in CKD mice compared to controls, and hypoxia did not induce HIF-2α activation in TECs in this study. Therefore, the role of HIF-2α overexpression in CKD still remains to be elusive. Finally, we did not evaluate the effect of administration of non-specific HIF stabilizers on CKD. Therefore, it is still uncertain that the findings in this study could be extrapolated to clinical relevance, and further studies are necessary whether HIF stabilizers are able to overexpress HIF-2α in TECs and whether overexpression of HIF-1α in TECs has similar effects to HIF-2α.

Taken together, renal tubular HIF-2α activation at the beginning of CKD aggravated renal fibrosis, whereas at a later stage of CKD, it inhibited the progression of renal fibrosis and improved renal function. Therefore, renal HIF-2α activation could represent a therapeutic target in late-stage CKD.

## Methods

### Animals

Animal care and use protocols for all experiments of this study were reviewed and approved by the IACUC at School of Medicine, Ewha Womans University (ESM 12-0201, ESM14-0265). All methods were performed in accordance with the relevant guidelines and regulations.

We used a transgenic mouse line containing hemagglutinin (HA)-tagged HIF2dPA, which escapes from recognition by pVHL owing to a proline to alanine substitution, preceded by a floxed stop codon cassette, rendering HIF-2α expression Cre-dependent^10^. Transgenic mice, which carried the three transgenes were generated by multiple breeding strategies (Fig. 1A), and progeny without all three transgenes were regarded wild-type. Given the large number of transgenes and complex breeding strategies, the genetic background strain for all mice used in this study was mixed. HIF-2α protein expression could be determined by western blotting or immunofluorescent staining with anti-HA antibody (sc-805; Santa Cruz Biotechnology Inc., Dallas, TX, USA) or anti-HIF-2α antibody (NB100-122; Novus biologicals Inc., Littelton, CO, USA). To obtain renal tubular cell-specific HIF-2α-overexpression at the time of choice, DOX (Sigma Chemical Co., St. Louis, MO, USA) was added to the drinking water at a concentration of 2 mg/mL for at least 3 days.

For the induction of renal fibrosis and CKD, the mice were fed a custom-made diet (Central Lab Animal Inc., Korea) containing 0.2% (w/w) adenine (A2786; Sigma Chemical Co.) for 2 or 4 weeks^32^. In addition, renal hypoxia measured by pimonidazole staining was effectively induced by 0.2% adenine-containing diet^33^. Transgenic and wild-type mice were divided into 6 experimental groups: (i) wild-type mice fed a standard diet (control), (ii) wild-type mice fed adenine-containing diet for 2 weeks, (iii) transgenic mice fed adenine-containing diet for 2 weeks with DOX administration at day 0, (iv) wild-type mice fed adenine-containing diet for 4 weeks, (v) transgenic mice fed adenine-containing diet for 4 weeks with DOX administration at day 0, and (vi) transgenic mice fed adenine-containing diet for 4 weeks with DOX administration at day 14. Mice were sacrificed at 2 or 4 weeks, and the kidneys were removed for histological evaluation and molecular analysis.

We detected quantitatively hypoxia gradients in animals with a hypoxyprobe™-1 (pimonidazole HCl; Hypoxyprobe Inc., Burlington, MA, USA) solution. Paraffin-embedded kidneys were stained with Masson’s trichrome for qualitative evaluation of renal fibrosis, and stained with Picro-Sirius Red (ab150681; Abcam, Cambridge, UK) for quantitative analysis of fibrosis. Then, we performed immunofluorescent staining with paraffin-embedded kidney sections, which were incubated with primary antibodies against hemagglutinin (sc-805; Santa Cruz Biotechnology Inc., Dallas, TX, USA), anti-HIF-2α antibody (NB100-122; Novus biologicals Inc., Littelton, CO, USA), and CD31 (MAB1398Z, EMD Millipore Co., Temecula, CA, USA).

### Isolation and Culture of Renal Tubular Epithelial Cells (TECs)

Primary renal TECs were isolated from PAX8-rtTA/tetO-Cre/HIF2dPA-HA transgenic male mice aged 4 weeks or less. For HIF-2α induction in cultured renal TECs, DOX was added into the media at a final concentration of 1 μg/mL or 5 μg/mL to reveal the dose-dependent HIF-2α effect. DOX (5 μg/mL) was also supplied into the media with the course of time (6-, 12-, 24-, and 48-hr) to show the time-dependent HIF-2α effect. In addition, hypoxia-exposed TECs were used to investigate endogenous HIF-2α expression and its relevant gene expressions.

### Molecular Analysis

We performed western blot analysis for the evaluation of fibronectin (rabbit, 1:1000; Dako), CD31 (rabbit; 1:1000; ab28364, Abcam), HIF-2α (rabbit; 1:500; NB100-122, Novus Biologicals Inc.), type 1 collagen (goat; 1:1000; 1310-01, Southernbiotech), p38 (rabbit; 1:1000, cell signaling), p-p38 (rabbit; 1:1000, cell signaling), and β-actin (mouse; 1:1000; A5441, Sigma Chemical Co.) protein expression. The relative mRNA expression levels were determined as ratios relative to control levels using quantitative reverse transcriptase polymerase chain reaction (RT-qPCR) analysis, and the sequences of primers used for the experiment are presented in Supplementary Table 1.

### Analysis in Human Subjects

Eight adult patients with IgA nephropathy (all male; median age, 49; median MDRD eGFR, 27.3 mL/min per 1.73 m^2^; median albumin-to-creatinine ratio, 568.7 mg/g) confirmed by renal biopsy were included. Kidney tissues were collected after obtaining the approval from the Institutional Review Board (IRB) of Yonsei University Heath System Clinical Trial Center (IRB No; 4-2006-0154), and all participants provided written informed consent.

We microdissected the renal tissue of patients, used tubulointerstitium for RT-qPCR analysis of a variety of relevant genes, and compared mRNA expression patterns according to stage of tubular atrophy/interstitial fibrosis (T stage) by the Oxford classification or according to the CKD stage based on the estimated glomerular filtration rate. The sequences of primers used for the experiment are presented in Supplementary Table 2.

### Equipment and Settings

Images of the stained tissues were captured under a polarized microscope (BX51 microscope; Olympus, Tokyo, Japan) and converted into gray-scale images for quantification. In addition, immunofluorescent mages were acquired using a confocal laser-scanning microscope (LSM5 PASCAL; Carl Zeiss Jena GmbH, Jena, Germany). And, all digitized images submitted with the final revision of the manuscript was 300 DPI. Moreover, the Anaeropack-anaero (< 1% O_2_ and 5% CO_2_; Mitsubishi Gas Chemical Co., Tokyo, Japan) was used to create a hypoxic condition. All images were submitted to Image J v1.49 (NIH, Bethesda, MD, USA) to quantify the percentage of fibrosis in total tissue within an image. Furthermore, all the methods were carried out in accordance with relevant guidelines and regulations, and all the experimental protocols were approved by School of Medicine, Ewha Womans University and Yonsei University Health System.

### Statistical Analysis

All values are expressed as the mean ± standard error of the mean (SEM). Statistical analysis was performed using PRISM 6.0 software (GraphPad, San Diego, CA, USA) and the statistic software SPSS, ver. 18.0 (SPSS Inc., Chicago, IL, USA). ANOVA with Fisher’s least significant difference (LSD) post hoc test was used for multiple comparisons. Student’s *t* test was performed to compare differences between two groups. *P*-values less than 0.05 were considered to be statistically significant.

## Electronic supplementary material

Supplementary Data
