# Supplementary material for: Selective tubular activation of hypoxia-inducible factor-2α has dual effects on renal fibrosis
Source: Sci Rep. 2017 Sep 12;7:11351. doi: 10.1038/s41598-017-11829-2 (PMC5596020; doi:10.1038/s41598-017-11829-2)
Supplement: Supplementary file 1 — Supplementary Data [file 41598_2017_11829_MOESM1_ESM.doc]

**Selective tubular activation of hypoxia-inducible factor-2α has dual effects on renal fibrosis**

Kyoung Hye Kong,^1†^ Hyung Jung Oh,^2†^ Beom Jin Lim,^3^ Minsuk Kim,^4^

Ki-Hwan Han,^4^ Youn-Hee Choi,^4,5^ Kihwan Kwon,^4^ Bo Young Nam,^3^

Kyoung Sook Park,^3^ Jung Tak Park,^3^ Seung Hyeok Han,^3^ Tae-Hyun Yoo,^3^

Shina Lee,^4^ Seung-Jung Kim,^4^ Duk-Hee Kang,^4^ Kyu Bok Choi,^4^ Vera Eremina,^6^ Susan E. Quaggin,^7^ Dong-Ryeol Ryu,^4,5^ and Shin-Wook Kang^3^

^1^Graduate School and ^2^Ewha Institute of Convergence Medicine, Ewha Womans University, Seoul, Korea;

^3^College of Medicine, Yonsei University, Seoul, Korea;

^4^School of Medicine, and ^5^Tissue Injury Defense Research Center, Ewha Womans University, Seoul, Korea;

^6^The Samuel Lunenfeld Research Institute, Toronto, Ontario, Canada;

^7^Feinberg Cardiovascular Research Institute and Division of Nephrology and Hypertension, Northwestern University, Chicago, Illinois, USA

**SUPPLEMENTARY DATA**

**
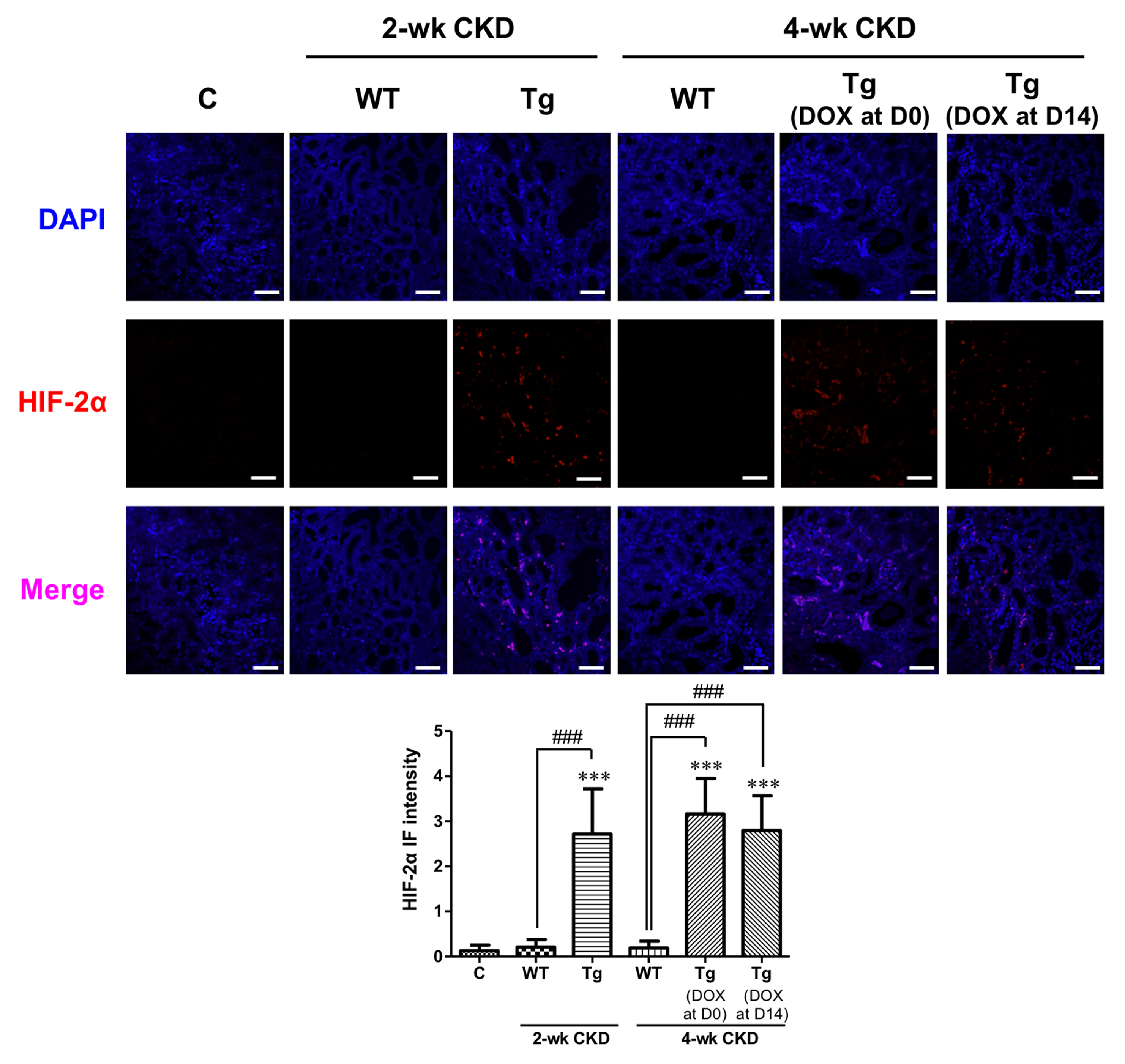
**

**Supplementary Figure S1.** Immunofluorescence staining for HIF-2α protein expression in wild-type and HIF-2α transgenic mice with chronic kidney disease (CKD) at 2 and 4 weeks. The expressions of HIF-2α were significantly upregulated in both 2- and 4-week transgenic mice compared with control and wild-type mice. Scale bars, 40 μm.

**
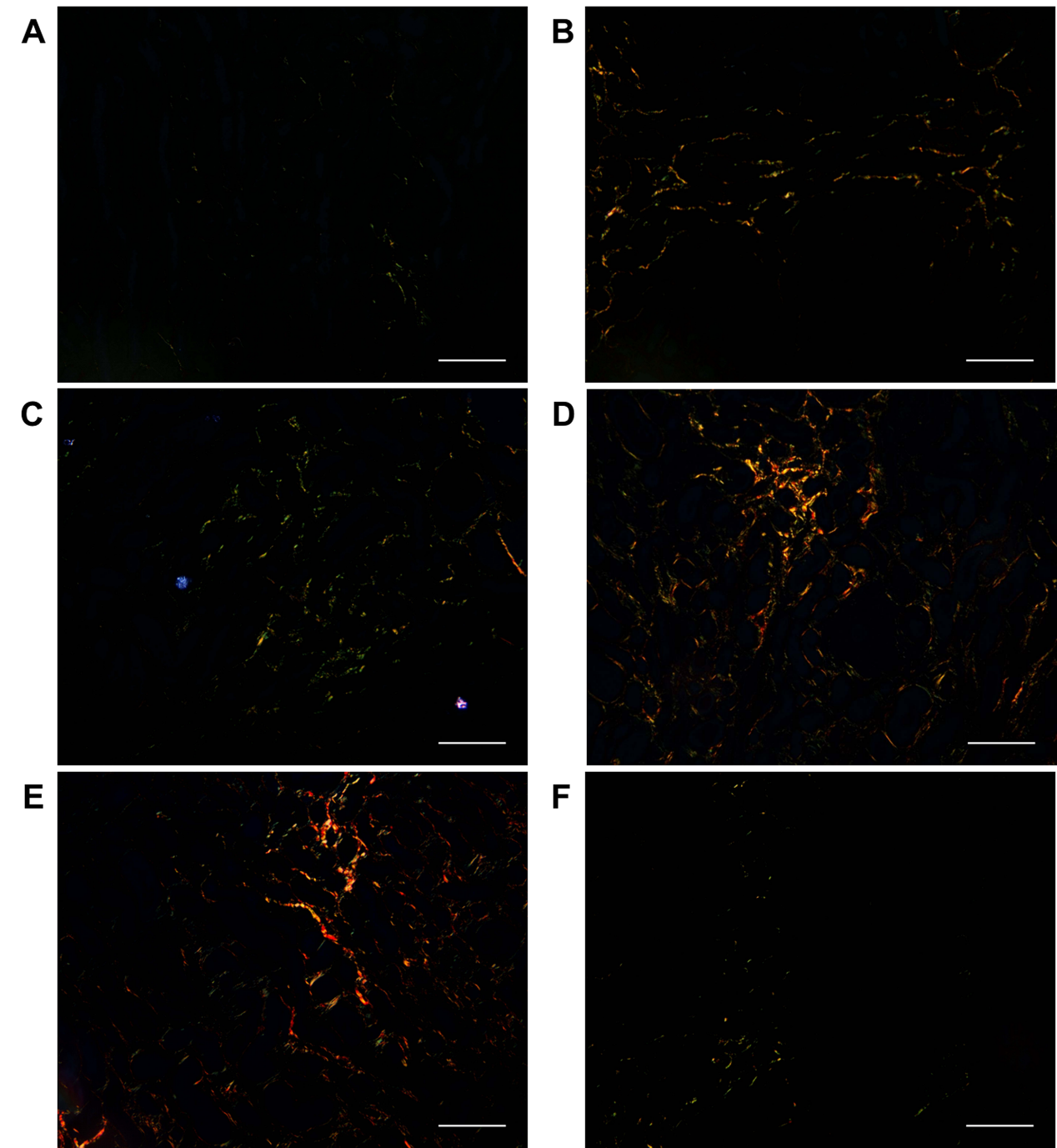
**

**Supplementary Figure S2.** Representative images of Sirius red staining showing the degree of renal fibrosis with polarization contrast illumination in (**A**) control mice, (**B**) wild-type CKD mice at 2 weeks, (**C**) HIF-2α transgenic CKD mice at 2 weeks, (**D**) wild-type CKD mice at 4 weeks, (**E**) HIF-2α transgenic CKD mice at 4 weeks with HIF-2α overexpression from D0, and (**F**) HIF-2α transgenic CKD mice at 4 weeks with HIF-2α overexpression from D14. CKD induction with a 0.2% adenine-containing diet for 2 weeks or for 4 weeks led to a significant increase in renal fibrosis. Increased renal fibrosis in wild-type CKD mice at 4 weeks was attenuated by HIF-2α activation at a later period, but was not significantly changed by HIF-2α activation from the beginning of CKD induction. Scale bars, 100 μm.


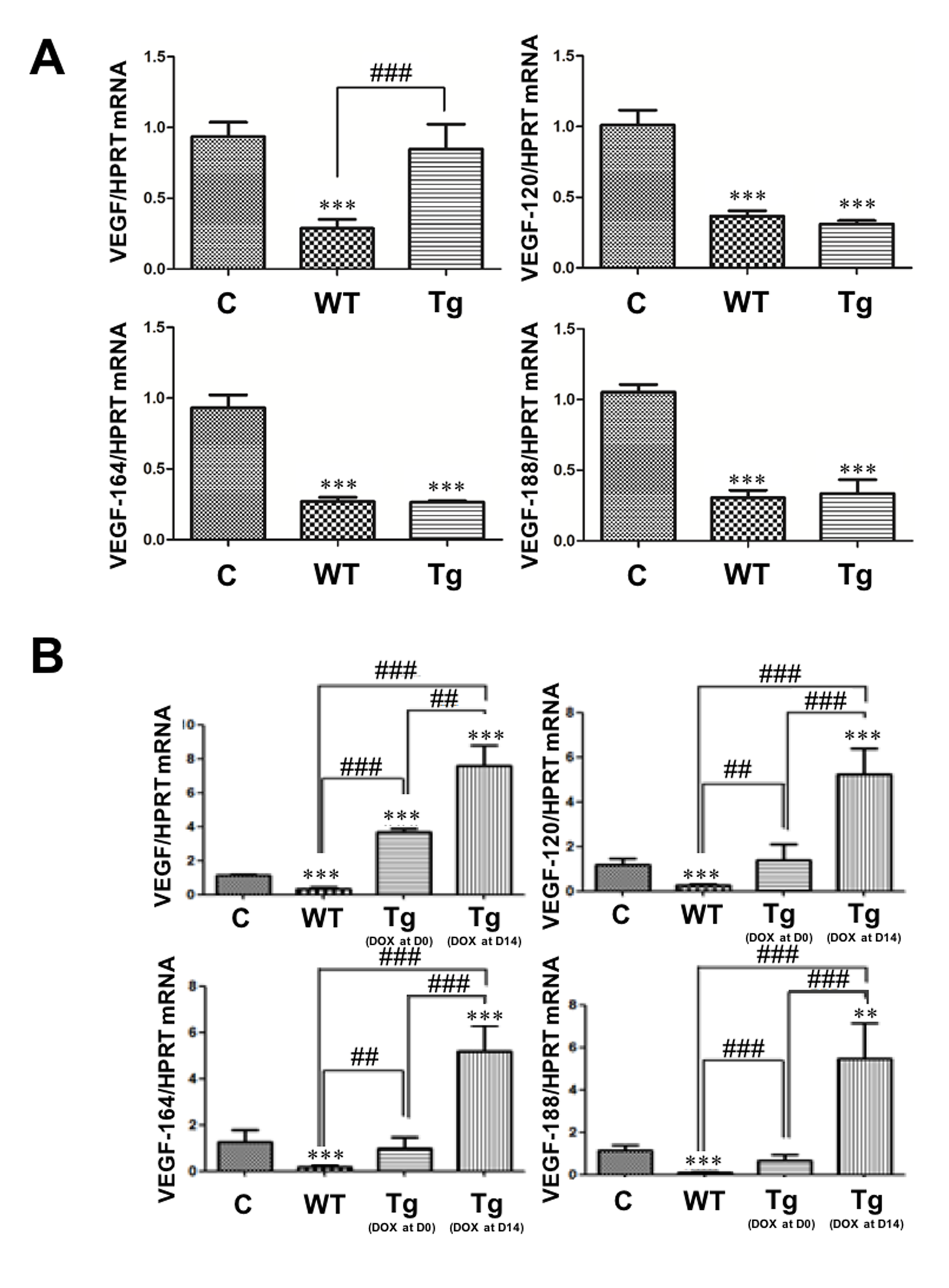


**Supplementary Figure S3.** The mRNA expressions of total vascular endothelial growth factor (VEGF) and its isoforms (120, 164, and 188). (**A**) Total VEGF mRNA expression was significantly lower in wild-type mice at 2 weeks after chronic kidney disease (CKD) induction compared with controls, whereas it was significantly higher in HIF-2α transgenic mice than that of wild-type mice. The mRNA expressions of VEGF isoforms were significantly lower in both wild-type and transgenic CKD mice than those of controls at 2 weeks. (**B**) At 4 weeks after CKD induction, mRNA expressions of total VEGF and its isoforms were significantly lower in wild-type CKD mice than those of controls, and they were higher in transgenic CKD mice than those of wild-type CKD mice. In addition, they were the highest in HIF-2α transgenic CKD mice at 4 weeks with HIF-2α overexpression from D14 among all groups. qPCRs were performed 3 to 4 times with RNA isolated lysates derived from 3 independent cell culture experiments. ***P* < 0.01, and ****P* < 0.001 *vs.* controls; #*P* < 0.05 , ##*P* < 0.01, and ###*P* < 0.001 between the two groups; bar graphs show mean ± SEM.

**
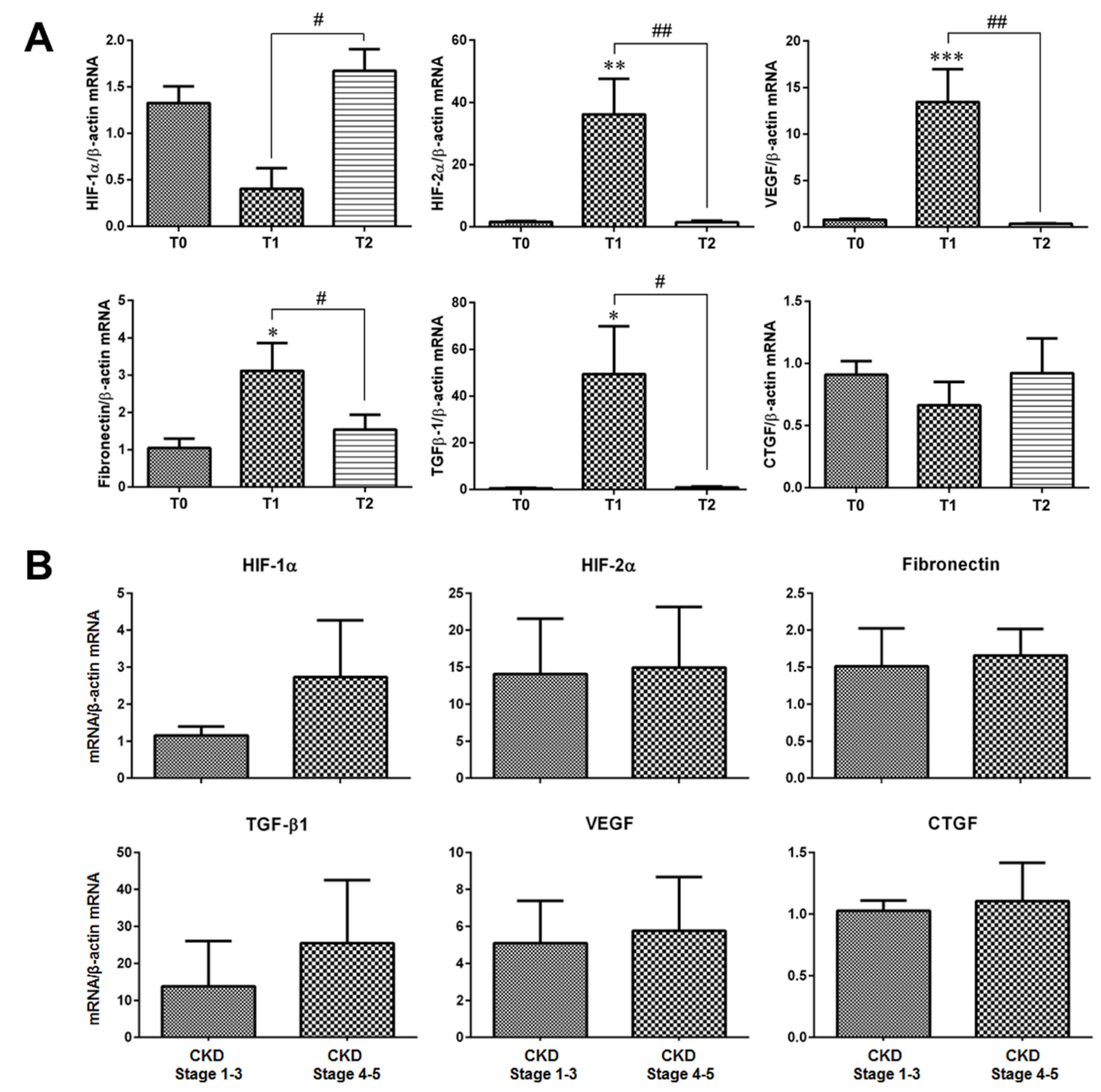
**

**Supplementary Figure S 4.** (A) The stage of tubular atrophy/interstitial fibrosis of the Oxford classification in human IgA nephropathy was associated with changes in the expression of a variety of genes. In patients with IgA nephropathy (n=8; all male; median age, 49; median MDRD eGFR, 27.3 mL/min per 1.73 m^2^; median albumin-to-creatinine ratio, 568.7 mg/g), tubulointerstitial tissues were used for RT-qPCR of relevant genes. When we divided the subjects according to the stage of tubular atrophy/interstitial fibrosis (T stage) by the Oxford classification, renal HIF-2α, VEGF, fibronectin, and TGF-β mRNA expression was significantly higher in stage T1 (n=3) than in stages T0 (n=3) and T2 (n=2). In contrast, HIF-1α mRNA expression was significantly lower in stage T1 patients than in stage T2 subjects. The mRNA expression of CTGF was not different among the three groups. (B) The relationship between CKD stages based on estimated glomerular filtration rate and the mRNA expression levels of various relevant genes. There were no significant differences in mRNA expression levels between CKD stage 1–3 patients (n=4) and CKD stage 4–5 patients (n=4). **P* < 0.05, ***P* < 0.01, and ****P* < 0.001 *vs.* stage T0; #*P* < 0.05 and ##*P* < 0.01 between the two groups; bar graphs show mean ± SEM.

**
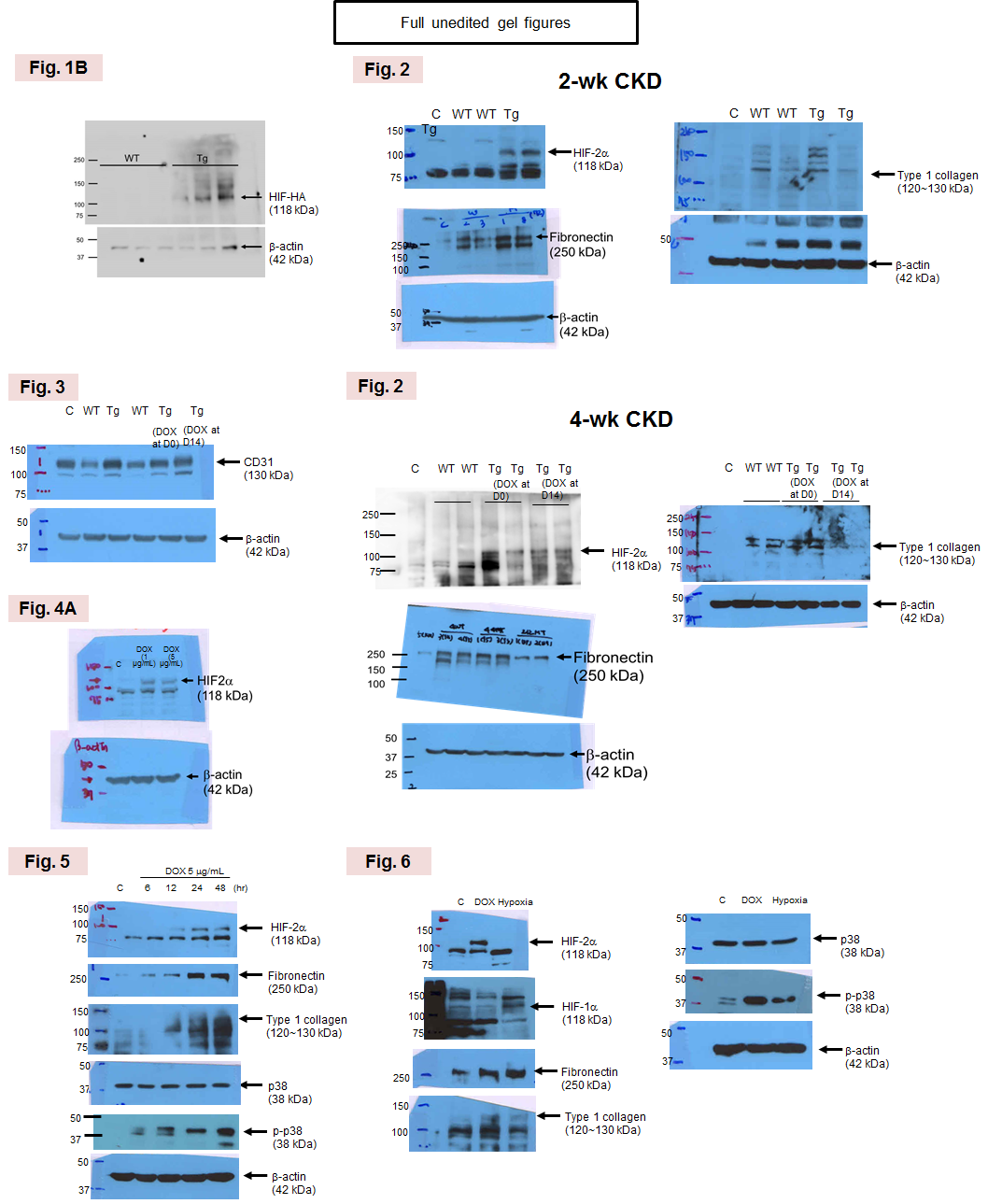
**

**Supplementary Figure S5. Full-length blots used in this study.**

**Supplementary Table S1. Sequential changes in body weights (g) in 2 week-CKD groups.**

| Groups | Body weight at baseline | Body weight  at 1 week | Body weight  at 2 weeks |
| --- | --- | --- | --- |
| WT; CKD | 29.9 ± 1.8 | 25.7 ± 0.8* | 23.5 ± 0.9* |
| HIF-2α Tg; CKD | 26.3 ± 1.1 | 23.6 ± 0.7*^,#^ | 22.3 ± 0.8* |
| HIF-2α Tg; No CKD | 30.4 ± 0.9 | 31.9 ± 1.4^#^ | 31.6 ± 1.3^#^ |

**P* < 0.05 and ***P* < 0.01 vs. the same group at baseline. There is no significant difference in body weight between WT CKD mice and HIF-2α Tg CKD mice.

**Supplementary Table S2. Sequential changes in body weights (g) in 4 week-CKD groups.**

| Groups | Body weight at baseline | Body weight  at 1 week | Body weight  at 2 weeks | Body weight  at 3 weeks | Body weight  at 4 weeks |
| --- | --- | --- | --- | --- | --- |
| WT; CKD | 33.6 ± 1.3 | 27.0 ± 1.6* | 24.7 ± 1.3* | 22.9 ± 1.1* | 23.5 ± 1.3* |
| HIF-2α Tg; CKD  (DOX at D0) | 30.9 ± 0.7 | 25.8 ± 1.1* | 23.3 ± 0.5* | 22.6 ± 0.6* | 21.3 ± 0.8* |
| HIF-2α Tg; CKD  (DOX at D14) | 27.2 ± 1.4^#^ | 24.1 ± 1.1* | 22.8 ± 1.0* | 21.6 ± 1.2* | 20.9 ± 1.0* |
| HIF-2α Tg; No CKD | 26.8 ± 0.6^#^ | 28.0 ± 0.3 | 30.3 ± 0.4*^,#^ | 31.7 ± 0.2*^,#^ | 33.5 ± 0.1*^,#^ |

**P* < 0.05 vs. the same group at baseline; ^#^*P* < 0.05 vs. WT CKD mice at the same period.

**Supplementary Table S3.** Sequences of mouse-specific primers used for RT-qPCR

| Gene | Forward | Reverse |
| --- | --- | --- |
| *Hif2a* | TTG ATG TGG AAA CGG ATG AA | GGA ACC TGC TCT TGC TGT TC |
| *Vegf* | CCA CGT CAG AGA GCA ACA TCA | TCA TCT CTC CTA TGT GCT GGC TTT |
| *Pai-1* | GGC TTC ATG CCC CAC TTC TTC | ATT CAC CAG CAC CAG GCG TGT |
| *Pgk1* | GGA AGC GGG TCG TGA TGA | GCC TTG ATC CTT TGG TTG TTT G |
| *Bnip3* | CAT GTC GCA GAG CGG GGA | GTC ACA GTG AGA ACT CTT G |
| *Fibronectin* | GAT GCA CCG ATT GTC AAC AG | ACT CTG ATC AGC ATG GAC CA |
| *Col1a1* | CCC GCC GAT GTC GCT AT | GCT ACG CTG TTC TTG CAG TGA T |
| *Lox* | CCA CAG CAT GGA CGA ATT CA | AGC TTG CTT TGT GGC CTT CA |
| *Hprt* | CAG ACT GAA GAG CTA CTG TAA TG | CCA GTG TCA ATT ATA TCT TCA AC |
| *Vegf120* | AAC GAT GAA GCC CTG GAG TG | TGA GAG GTC TGG TTC CCG A |
| *Vegf164* | AAC GAT GAA GCC CTG GAG TG | GAC AAA CAA ATG CTT TCT CCG |
| *Vegf188* | AAC GAT GAA GCC CTG GAG TG | AAC AAG GCT CAC AGT GAA CG |

**Supplementary Table S4.** Sequences of human-specific primers used for RT-qPCR

| Gene | Forward | Reverse |
| --- | --- | --- |
| *Hif2a* | GTG ACA TGA TCT TTC TGT CGG AA | CGC AAG GAT GAG TGA AGT CAA A |
| *Hif1a* | TGC AGA ATG CTC AGA GAA AGC GAA | GCT GCA TGA TCG TCT GGC TGC T |
| *Ctgf* | GTG CAC CGC CAA AGA TGG T | AAG GAC TCT CCG CTG CGG TA |
| *Vegf* | TCT GCT GTC TTG GGT GCA TT | GCA GTA GCT GCG CTG ATA GA |
| *Tgfb1* | TGG AAG TGG ATC CAC GCG CCC AAG G | GCA GGA GCG CAC GAT CAT GTT GGA C |
| *Fibronectin* | GGT GAC ACT TAT GAG CGT CCT AAA | AAC ATG TAA CCA CCA GTC TCA TGT G |
| *Actb* | GTC ACC AAC TGG GAC GAC A | TGG CCA TCT CTT GCT CGA A |

**SUPPLEMENTARY METHODS**

**Animals**

PAX8-rtTA, tetO-Cre, HIF1dPA-HA and HIF2dPA-HA transgenic mice were obtained from the Samuel Lunenfeld Research Institute at Mount Sinai Hospital, Toronto, ON, Canada. Transgenic mice carrying the three transgenes were generated by multiple breeding strategies (Fig. 1A), and progeny without all three transgenes were regarded wild-type. Mice carrying the HIF2dPA-HA transgene were bred with mice with the PAX8-rtTA driver to allow overexpression of HIF-2α in renal tubular cells^1^. A Lox-stop-Lox cassette was introduced at the 5′ end of the HIF2dPA transgene cDNA to allow transcription to be dependent on Cre-mediated excision of the stop element^2^. In addition, since a hemagglutinin (HA) tag was expressed upon translation of the transgene, HIF-2α protein expression could be determined by western blotting or immunofluorescent staining with anti-HA antibody (sc-805; Santa Cruz Biotechnology Inc., Dallas, TX, USA) (Fig. 2A, B). In addition, we performed immunofluorescent staining with anti-HIF-2α antibody (NB100-122; Novus biologicals Inc., Littelton, CO, USA).

Genotypes were confirmed by PCR using tail genomic DNA. The following primers were used for genotyping: PAX8-rtTA, 5′-CGT AGG AAA GCT GCG AGT GT-3′, 5′-AGT GGG TAT GAT GCC TGT CC-3′; tetO-Cre, 5′-AGA GTC ATC CTT AGC GCC GTA AAT CAA T-3′, 5′-GTG CAA GTT GAA TAA CCG GAA ATG-3′; and HA-HIF2dPA, 5′-GAA ATC AGC TTC CTG CGA AC-3′, 5′-TCA TGA AGA AGT CCC GCT CT-3′.

Given the large number of transgenes and complex breeding strategies, the genetic background strain for all mice used in this study was mixed.

**Animal Experiments**

Six- to eight-week old male mice weighing 25–35 g were used for all animal experiments and were allowed food and water *ad libitum*. For the induction of renal fibrosis and CKD, the mice were fed a custom-made diet (Central Lab Animal Inc., Korea) containing 0.2% (w/w) adenine (A2786; Sigma Chemical Co.) for 2 or 4 weeks^3^.

All mice were anesthetized with an intraperitoneal injection of a mixture of zoletil and rompun (30 mg/kg of zoletil, Virbac Laboratories, Carros, France; 20 mg/kg of rompun, Bayer Korea, Ansan, Korea) and were sacrificed at 2 or 4 weeks. The kidneys were removed for histological evaluation and molecular analysis.

**Serum Chemistry Analysis**

Blood was drawn via cardiac puncture at the sacrifice. Serum was prepared from blood collected in serum-separating tubes and frozen at -80°C until use. Serum creatinine (Cr) and blood urea nitrogen (BUN) were determined using an automated analyzer for routine chemistry at the Seoul Medical Science Institute.

**Pimonidazole Staining**

For quantitative detection of hypoxia gradients in animals, we intraperitoneally administered a hypoxyprobe™-1 (pimonidazole HCl; Hypoxyprobe Inc., Burlington, MA, USA) solution at a dose of 60 mg/kg body weight. Mice were euthanatized 90 min after hypoxyprobe™-1 injection. The kidneys were excised, paraffin-embedded, and sectioned. The sections were deparaffinized and treated with 3% hydrogen peroxide in distilled water for 5 min. After washing, the sections were blocked with 1% bovine serum albumin (BSA) in Tris-buffered saline (TBS), and incubated with fluorescein isothiocyanate (FITC)-conjugated mouse IgG1 monoclonal antibody (FITC-MAb1, 1:100) overnight at 4°C. Then, the sections were washed with TBS and incubated with rabbit anti-FITC-conjugated antibody and horseradish peroxidase as a secondary reagent (1:100) for 30 min at room temperature. After washing, they were treated with diaminobenzidine solution for 5 min.

**Histology**

Mice were anesthetized and perfused intracardially with normal saline (0.9% NaCl) and then with 4% paraformaldehyde (PFA) in 0.1 M phosphate-buffered saline (PBS, pH 7.4). After perfusion, the kidneys were removed, placed in 4% PFA at 4°C overnight, and embedded in paraffin. Paraffin-embedded kidneys were sectioned at a thickness of 4 μm, and stained with Masson’s trichrome for qualitative evaluation of renal fibrosis. For quantitative analysis of fibrosis, sections of kidney were stained with Picro-Sirius Red (ab150681; Abcam, Cambridge, UK). Briefly, Sirius Red staining was performed by incubating slides in 0.1% Sirius Red F3B for 1 hr, washing twice in acidified water, dehydrating thrice in 100% ethanol, and finally, clearing in xylene. Images of the stained tissues were captured under a polarized microscope and converted into gray-scale images for quantification. Sirius red fibrosis was quantified over the entire kidney section using 10 non-overlapping 10× images as described previously^4^. All images were submitted to Image J v1.49 (NIH, Bethesda, MD, USA) to quantify the percentage of fibrosis in total tissue within an image.

**Immunofluorescent Staining**

Paraffin-embedded kidney sections were deparaffinized and then treated with 3% hydrogen peroxide and 10% methanol in PBS for 10 min to block endogenous peroxidase activity. The sections were treated with 0.1% Triton X-100, 2% BSA, and then blocked with 2% normal horse serum in PBS. The sections were incubated with primary antibodies against HA (sc-805; Santa Cruz Biotechnology Inc., Dallas, TX, USA), anti-HIF-2α antibody (NB100-122; Novus biologicals Inc., Littelton, CO, USA), Cytokeratin 18 (ab668; Abcam, Cambridge, UK), ~~ZO-1 (40-2300, Invitrogen, Carlsbad, CA, USA),~~ and CD31 (MAB1398Z, EMD Millipore Co., Temecula, CA, USA) overnight at 4°C. Then, they were washed with PBS and incubated with anti-rabbit IgG labeled with Alexa Fluor-594 for HA and anti-HIF-2α antibody, anti-mouse IgG labeled with Alexa Fluor-488 for cytokeratin 18 ~~and ZO-1~~, and anti-hamster IgG labeled with Alexa Fluor-568 for CD31 secondary antibodies for 1 hr at room temperature, washed, stained with DAPI, and mounted using DAKO mounting medium (S3023; DAKO Denmark, Glostrup, Denmark). Moreover, we measured the intensity of HIF-2α-stained area with Image J v1.49 (NIH, Bethesda, MD, USA) and compared the ratios of its expression between wild-type and transgenic mice. Images were acquired using a confocal laser-scanning microscope (LSM5 PASCAL; Carl Zeiss Jena GmbH, Jena, Germany).

**Isolation and Culture of Renal Tubular Epithelial Cells (TECs)**

Primary renal TECs were isolated from PAX8-rtTA/tetO-Cre/HIF2dPA-HA transgenic male mice aged 4 weeks or less. After the mice were anesthetized, the kidneys were immediately excised, dissected visually, placed in 1 ml of ice-cold Dulbecco’s phosphate-buffered saline (DPBS), and minced into pieces of less than 1 mm^3^. Fragments were transferred to collagenase I solution (1 mg/ml in DPBS, 9001-12-1; Gibco-Invitrogen, Carlsbad, CA, USA) and digested for 30 min at 37°C. Then, the supernatant was resuspended in RPMI 1640 medium and sieved through a 100-μm nylon mesh and centrifuged at 3000 rpm for 10 min. The pellet was resuspended in sterile red blood cell lysis buffer (8.26 g NH_4_Cl, 1 g KHCO_3_, 0.037 g EDTA/L ddH_2_O) and kept on ice for 3 min. The pellet was washed twice with collagenase 1 solution consisting of 10% FBS, 1% penicillin-streptomycin, and 20 ng/mL of epithelial growth factor (EGF; Sigma Chemical Co.)^5^. After isolating and seeding the primary TECs from live mice, it took about 7 days until the cells were 70% confluent in the plate. At this time, we administered DOX into the media for the treated groups or not for the controls. And then, unless we mentioned the harvest time specifically in the text, the cells were harvested after 48 hours at the time of plates with around 100% cell-confluency.

For HIF-2α induction in cultured renal TECs, DOX was added into the media at a final concentration of 1 μg/ml or 5 μg/ml^6^. At 48 hr after medium change, the cells were harvested for the following experiments, and the cell proliferation rate was measured to evaluate the cytotoxicity of DOX using the CellTiter 96 AQueous One Solution Cell Proliferation Assay kit (G3580; Promega Co., Madison, WI, USA) according to the manufacturer’s protocol. After experimental treatments, 20 μl of 3-(4,5-dimethylthiazol-2-yl)-5-(3-carboxymethoxyphenyl)-2-(4-sulfophenyl)-2H tetrazolium (MTS) reagent was added to each well of the 96-well plate containing samples in 100 μl of culture medium, and then incubated at 37°C for 4 hrs. After incubation, the absorbance at 490 nm was measured using a microplate reader (Molecular Devices, Spectra MAX, Sunnyvale, CA, USA). Moreover, DOX (5 μg/ml) was also supplied into the media with the course of time (6-, 12-, 24-, and 48-hr), and the cells were harvested with above same methods. To create a hypoxic condition, the Anaeropack-anaero (<1% O_2_ and 5% CO_2_; Mitsubishi Gas Chemical Co., Tokyo, Japan) was used. According to the manufacturer’s protocol, the cultured renal TECs were placed at anaerobic jar and removed the paper sachet out of the foil bag and then immediately placed the paper sachet into an anaerobic jar and closed the jar lid. The cultured renal TECs were cultured under hypoxic condition for 48hr^7^.

**Western Blot Analysis**

Homogenized whole kidney and cultured TECs were resuspended in protein extraction solution (Pro-Prep™; iNtRON, SungNam, Korea) and incubated at -20°C for 20 min. The suspension was centrifuged at 16,000 rpm at 4°C for 30 min, and the supernatant was transferred to e-tubes for western blot analysis. The protein concentration in each sample was measured using a Pierce® BCA protein assay kit (Thermo Fisher Scientific Inc., Rockford, IL, USA). For the detection of nuclear hemagglutinin protein, the nuclear and cytoplasmic fractions were separated using the FractionPREP Cell Fractionation Kit (BioVision Inc., Mountain View, CA, USA) according to the manufacturer’s instructions. Briefly, the kidney was lysed at 4°C in cytosol buffer (with protease cocktail and dithiothreitol). After pestle homogenization for 30 s, the lysates were centrifuged at 700 × *g* for 7 min, and the supernatant (cytoplasmic fraction) was stored at -80°C until required. The pellet in nuclear buffer (with protease cocktail and dithiothreitol) was homogenized using a pestle homogenizer for 30 s. After homogenization, the samples were vortexed for 15 s every 10 min, and then kept on ice for 20 min. Then, the lysates were centrifuged at 3000 × *g* for 5 min, and the supernatant was harvested as the nuclear fraction.

Equal amounts of protein for each sample were lysed in sodium dodecyl sulfate (SDS) sample buffer [2% SDS, 10 mM Tris-HCl, pH 6.8, 10% (v/v) glycerol], treated with Laemmli sample buffer, heated at 100°C for 5 min, and electrophoresed in an 8% SDS-polyacrylamide gel. The proteins were electrophoretically transferred to an Immun-Blot© PVDF membrane (Cat. #162-0177; BioRad Laboratories Inc., Hercules, CA, USA). The membrane was placed immediately into a blocking solution (5% BSA) at room temperature for 30 min, and then incubated with diluted primary antibodies against fibronectin (rabbit, 1:1000; Dako) and β-actin (mouse; 1:1000; A5441, Sigma Chemical Co.) in TBS-T buffer (10 mM Tris, 100 mM NaCl, 0.2% Tween-20, pH 7.5) at 4°C for overnight. On the next day, the membrane was washed in TBS-T buffer, incubated with secondary polyclonal anti-mouse antibody or polyclonal anti-rabbit antibody (1:10000; Santa Cruz Biotechnology) in TBS-T buffer at room temperature for 1 hr. Horseradish-conjugated secondary antibody labeling was detected using enhanced chemiluminescence reagent (ECL; Thermo Scientific Pierce, Rockford, IL, USA) by exposure to an X-ray film. The band densities were measured using ImageJ v1.49 (NIH, Bethesda, MD, USA). Moreover, we complied with the digital image and integrity policies (http://www.nature.com/srep/journal-policies/editorial-policies#digital-image).

**Quantitative Reverse Transcriptase Polymerase Chain Reaction (RT-qPCR) Analysis**

Total RNA was prepared from mouse kidneys and from primary cultures of renal TECs using Easy-BLUE isolation reagent (iNtRON Biotechnology, Daejeon, Korea) according to the manufacturer’s protocol. cDNA was synthesized from 2 μg of purified total RNA using M-MLV reverse transcriptase (Enzynomics, Seoul, Korea). RT-qPCR was performed using the ABI StepOne Real-Time PCR system (Applied Biosystems, Foster City, CA, USA) with the QuantiFast SYBR green PCR kit (Qiagen Inc., Hilden, Germany) according to general protocols.

Each sample was analyzed in triplicate for each gene. Expression levels were normalized to that of hypoxanthine guanine phosphoribosyl transferase (HPRT). The relative mRNA expression levels were determined as ratios relative to control levels using the 2^-ΔΔCT^ method. The sequences of primers used for the experiment are presented in Supplemental Table 1. We examined the melting curve to verify that a single product was amplified.

**REFERENCES**

1 Traykova-Brauch, M. *et al.* An efficient and versatile system for acute and chronic modulation of renal tubular function in transgenic mice. *Nat Med* **14**, 979-984, doi:10.1038/nm.1865 (2008).

2 Kim, W. Y. *et al.* Failure to prolyl hydroxylate hypoxia-inducible factor alpha phenocopies VHL inactivation in vivo. *EMBO J* **25**, 4650-4662, doi:10.1038/sj.emboj.7601300 (2006).

3 Hamasaki, Y. *et al.* A 5-hydroxytryptamine receptor antagonist, sarpogrelate, reduces renal tubulointerstitial fibrosis by suppressing PAI-1. *Am J Physiol Renal Physiol* **305**, F1796-1803, doi:10.1152/ajprenal.00151.2013 (2013).

4 Street, J. M. *et al.* Automated quantification of renal fibrosis with Sirius Red and polarization contrast microscopy. *Physiol Rep* **2**, doi:10.14814/phy2.12088 (2014).

5 Han, S. H. *et al.* Deletion of Lkb1 in Renal Tubular Epithelial Cells Leads to CKD by Altering Metabolism. *J Am Soc Nephrol* **27**, 439-453 doi:10.1681/ASN.2014121181 (2016).

6 Jechlinger, M., Podsypanina, K. & Varmus, H. Regulation of transgenes in three-dimensional cultures of primary mouse mammary cells demonstrates oncogene dependence and identifies cells that survive deinduction. *Genes Dev* **23**, 1677-1688, doi:10.1101/gad.1801809 (2009).

7 Higashikuni Y *et al.* The ATP-binding cassette transporter BCRP1/ABCG2 plays a pivotal role in cardiac repair after myocardial infarction via modulation of microvascular endotherlial cell survival and function. *Arterioscler Thromb Vasc Biol* **30,** 2128-2135, doi:10.1161/ATVBAHA.110.211755 (2010).
